# Prenatal exposure to persistent organic pollutants and metals and problematic child behavior at 3–5 years of age: a Greenlandic cohort study

**DOI:** 10.1038/s41598-021-01580-0

**Published:** 2021-11-12

**Authors:** Simon Kornvig, Maria Wielsøe, Manhai Long, Eva Cecilie Bonefeld-Jørgensen

**Affiliations:** 1 Center for Arctic Health and Molecular Epidemiology, Department of Public Health, Aarhus University, Aarhus, Denmark; 2 Greenland Centre for Health Research, University of Greenland, Nuuk, Greenland

**Keywords:** Developmental neurogenesis, Neurological disorders, Developmental biology

## Abstract

High levels of persistent organic pollutants (POPs) and heavy metals are found in Arctic populations. POP and heavy metals are linked to impaired cognitive development. This study examined associations between prenatal POP and metals exposure and problematic child behavior using the Strength and Difficulties Questionnaire (SDQ). POPs and metals were measured in 102 pregnant Greenlandic women. During follow-up at 3–5 years, parents answered an assisted questionnaire including children’s SDQ scores. Associations were analyzed using linear and logistic regression analyses and adjusted for maternal plasma cotinine, educational level and age at delivery. In the adjusted analyses, the medium tertile of hexachlorobenzene (β = 3.06, *p* = 0.010), β-hexachlorocyclohexane (β = 3.58, *p* = 0.004) and trans-nonachlor (β = 2.06, *p* = 0.082) were positively associated with SDQ scores. The continuous cis-nonachlor (OR = 1.09, *p* = 0.079), dichloro-diphenyl-dichloroethylene (OR = 1.01, *p* = 0.077), trans-nonachlor (OR = 1.01, *p* = 0.091), and sum Organochlorine-Pesticides (OR = 1.00, *p* = 0.094) were positively associated with abnormal SDQ score and the continuous mirex (OR = 1.28, *p* = 0.096), oxychlordane (OR = 1.04, *p* = 0.066), and trans-nonachlor (OR = 1.02, *p* = 0.071) with abnormal hyperactivity score. We found no consistent evidence of associations between polychlorinated biphenyls, perfluoroalkylated substances and heavy metals and problematic behavior. Prenatal organochlorine pesticide exposure associated significantly with problematic behavior in 3–5 year old children.

## Introduction

Persistent organic pollutants (POPs) are found ubiquitously in the environment. They originate from the worldwide industry and are resistant to degradation through natural, chemical and biological processes. POPs include lipophilic POPs e.g. *polychlorinated biphenyls (PCBs)*, *organochlorine pesticides (OCPs)* and the amphiphilic *perfluoroalkylated substances (PFASs)*. Other environmental pollutants of concern are heavy metals, such as mercury (Hg), lead (Pb) and cadmium (Cd), which are also widespread in the environment.

At certain concentrations both POPs and heavy metals are considered toxic and several studies found them in living organisms—including humans^1^. POPs can decrease the body's defense against oxidative stress and are classified as endocrine-disrupting chemicals (EDCs). Some EDCs are shown to interact with e.g. the estrogen and androgen receptors and thus modulating their signaling^2,3^. In addition, studies show that POPs can disrupt thyroid hormones in rodent’s such as mice and rats^1,4–6^ as well as in human^7,8^. Heavy metals are considered systemic toxicants known to affect and damage multiple organ systems even at lower levels of exposure and some of them are classified as ‘known’ and ‘probable’ human carcinogens^9^.

Political actions over the last decades through regulation and health interventions have had an effect on the exposure levels of the lipophilic POPs and heavy metals. Studies showed decreasing levels in both environment and humans for the regulated POPs^10–13^. Unfortunately, some unregulated POPs such as e.g. PFASs are still increasing^14^. Both POPs and heavy metals are found widespread in the environment with continuous exposure to humans^14–16^.

Globally, the Arctic area has the highest concentrations of POPs and heavy metals^1^. The pollutants are transported though sea-currents and atmospheric movement to the Arctic region and bio-accumulate in the wildlife animals and humans^17–25^. The exposure pathway of POPs and metals for the general population is mainly oral (food intake and drinking water). Due to the lipophilic property, PCBs, OCPs and Hg are stored in fat rich tissues, whereas the PFAS bind to proteins and Pb are mainly found in organs. With respect Cd, the exposure are both oral and via smoking^1^. The Greenlandic Inuit, still to some extent, rely on traditional food sources from the marine food chain, e.g. marine mammals, large predator fish and fish in general, thus, they are particularly exposed to POPs and heavy metals. However, in the recent 30–50 years, a gradually transition from a traditional Greenlandic diet to a more western diet has been observed^26–28^. The transition has resulted in a decreased exposure to some POPs and heavy metals, but has increased the average body mass index (BMI) and serum triglycerides, which possesses new health problems^29^.

The exposure to POPs and heavy metals is of particular concern during pregnancy. Several studies showed that the pollutants can cross the placenta barrier^30–32^ and a negative correlation between prenatal exposure and fetal growth and development has been shown^33–36^. Also, the exposure to heavy metals poses health risks to the developing fetus as it is in its most vulnerable state^37–39^. Studies show examples of negative effects of POP exposure e.g. immune disruption, reduced reproductive ability, cancer, and metabolic defects, poor gross motor function as well as child cognitive development^4,5,40–45^. Other studies point to the negative effects of heavy metals exposure e.g. allergies, endocrine disorders, risk of cancer, neurological diseases, and decreased cognition^46–48^.

Several studies have focused on prenatal exposure to POPs and heavy metals and neuro-developmental deficits during childhood and for instance problematic behavior or hyperactivity. The studies are, however, inconclusive, since some show associations between pollutant exposure and problematic behavior^49–58^ and others do not^59–63^.

The present study is part of the ACCEPT (Adapting to Climate Change, Environmental Pollution and Dietary Transition) birth cohort (2010–2015)^27,28^. Of the 614 pregnant women included, 102 families (mothers, fathers and children) were followed up 3–5 years after birth. The aim of this study was to examine the associations between prenatal exposure to a wide range of POPs and metals and problematic child behavior at 3–5 years of age measured using the Strength and Difficulties Questionnaire (SDQ). We hypothesized that prenatal POP and metal exposure was associated with Problematic Child Behavior (high/abnormal SDQ score and hyperactivity score) measured using the SDQ.

## Materials and methods

### Study population

This follow-up study is based on the ACCEPT mother–child cohort established in Greenland with the overall aim of exploring health and dietary changes during a period of lifestyle transition and climate change^27,28^.

**Figure 1:**
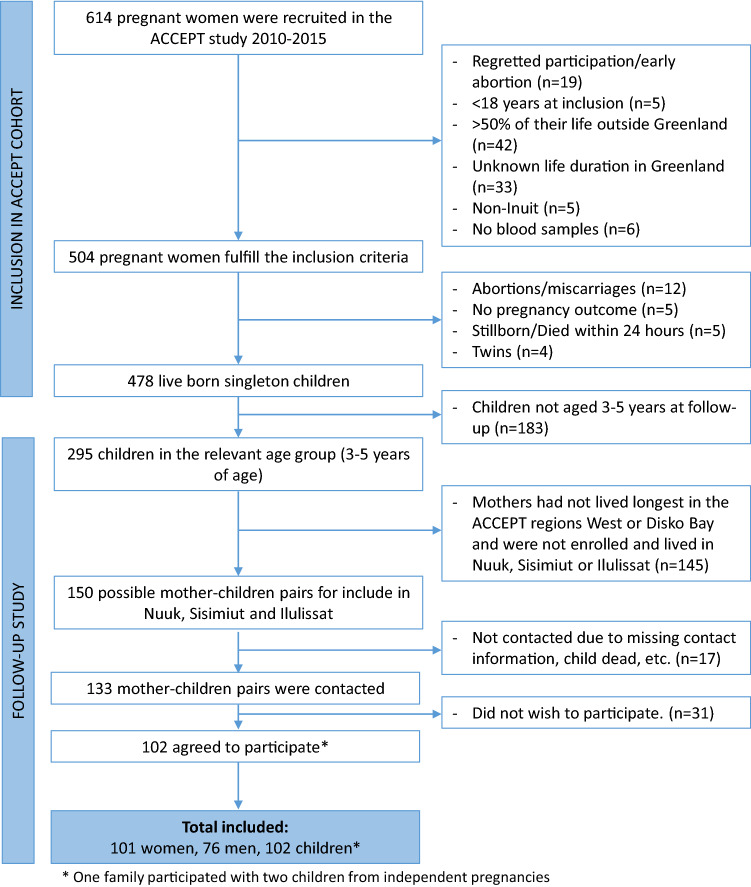
Flowchart showing the process of inclusion and exclusion of mother–child pairs for the ACCEPT follow-up study.

In total 614 pregnant Greenlandic women were recruited by a clinical doctor between August 2010 and 2011 and by midwives between June 2013 and September 2015. The women were recruited from 16 towns distributed over five ACCEPT defined Greenlandic regions^27,28^. To be included in the project, the women must have lived more than 50% of their lives in Greenland, be over the age of 18 at the time of inclusion, and be of Greenlandic descents (having at least one Inuit parent or speak Greenlandic as their native language). Amongst the 614 enrolled women, 110 were excluded due to early abortion/withdrawal of consent (n = 19), < 18 years at enrollment (n = 5), more than 50% of their time spent outside Greenland (n = 42), no information on life duration in Greenland (n = 33), being non-Inuit (n = 5) or no blood samples (n = 6) (Fig. 1).

Therefore, 504 of the enrolled women fulfilled the inclusion criteria and of those 478 gave birth to a live born singleton baby with birth outcome available. At the time of follow-up (May 2019–January 2020), 295 live born singleton children were in the relevant age of 3–5 years (children of mothers recruited in 2013–2015). Of these, 150 fulfilled the follow-up criteria (mothers had lived longest in the ACCEPT defined regions, West or Disko Bay, and resided in Nuuk, Sisimiut, and/or Ilulissat). We contacted 133 ACCEPT mothers, and 102 agreed to participate (Fig. 1). In total, 102 children, 101 mothers, and 76 fathers ended up participating in the follow-up (one family participated with two children from independent pregnancies). The participation rate at follow-up was 76.6%, and those who did not accept to participate mainly gave lack of time as the reason.

After receiving a detailed description of the ACCEPT project, all participants gave informed consent to participate. They were informed that they could withdraw their consent at any time in the process and that their participation was completely voluntary^27,28,64^. The present study has been conducted in accordance with the Helsinki Declaration and has been approved by the Ethical Committee for Scientific Investigations in Greenland.

### Maternal data collection at pregnancy

Maternal pregnancy lifestyle data was collected from medical records and questionnaires at inclusion, including age at delivery (year), pre-pregnancy body mass index (BMI, kg/m^2^), parity, educational level (primary school, high school, technical college, university), alcohol consumption status before and during pregnancy (< 1 time a month, 1 time a month, 2–3 times a month, > 1 time a week) and smoking history (never, former, current). The overall format of the questionnaire had been used in the previous international project INUENDO^52,65^ and the ACCEPT study^27,28^. From the Chief Medical Office, data on birth outcomes were obtained: gestational age at birth (GA), birth weight, birth length and birth head circumference. These measurements were done by midwifes.

Venous blood samples from the pregnant women were taken at the inclusion in the ACCEPT study. All 102 maternal blood samples in this study were collected in the first trimester before the end of week 13. The blood samples were stored at − 80 °C until analyses.

#### Serum lipophilic POP concentrations

Le Centre de Toxicologie du Québec of Canada, a certified laboratory by the Canadian Association for Environmental Analytical Laboratories analyzed the serum samples for 35 lipophilic POPs and lipids^66^. The 11 organochlorine pesticides (OCPs) [p,p-DDT (dichlorodiphenyltrichloroethane), p,p’-DDE (dichlorodiphenyldichloroethylene), aldrin, mirex, β-hexachlorocyclohexane (β-HCH), hexachlorobenzene, cis- and trans-nonachlor, and α-, γ- and oxy-chlordane], 14 polychlorinated biphenyl (PCB) congeners [PCB 28, 52, 99, 101, 105, 118, 128, 138, 153, 156, 170, 180, 183, 187], and 10 flame retardants including one polybrominated biphenyl [PBB153] and nine polybrominated diphenyl ethers (PBDEs) [PBDE 15, 17, 25, 28, 33, 47, 99, 100, 153] were analyzed in purified extracts by high-resolution gas chromatography with electron capture detection. Serum total cholesterol (TC), free cholesterol (FC), triglycerides (TG) and phospholipids (PL) were determined and serum total lipids were calculated using formation: 1.677 (TC-FC) + FC + TG + PL^66^. All determined PCBs, OCPs and PBDEs were adjusted to the serum lipid content and reported as μg/kg serum lipid. If the value was below the detection limit (DL), we assigned the value given by the DL divided by two (DL/2).

#### Serum PFAS concentrations

The Department of Environmental Science, Aarhus University, Denmark analyzed the serum samples for the 16 PFAS. The perfluorinated sulfonic acids (PFSAs): Perfluorobutanesulfonic acid (PFBS, C4), perfluorohexane sulfonate (PFHxS, C6), perfluoroheptanesulfonate (PFHpS, C7), perfluorooctane sulfonate (PFOS, C8), perfuoro-1-decanesulfonate (PFDS, C10), perfluorooctane sulfonamide (PFOSA, C8), and the perfluorinated carboxylic acids (PFCAs): perfluoropentanoic acid (PFPeA, C5), perfluorohexanoic acid (PFHxA, C6), perfluoroheptanoic acid (PFHpA, C7), perfluorooctanoic acid (PFOA, C8), perfluorononanoic acid (PFNA, C9), perfluorodecanoic acid (PFDA, C10), perfluoroundecanoic acid (PFUnA, C11), perfluorododecanoic acid (PFDoA, C12), perfluorotridecanoic acid (PFTrA, C13) and perfluorotetradecanoic acid (PFTeA, C14) measured using liquid chromatography-tandem mass spectrometry with electrospray ionization^67^. If the value was below the DL, the value was given by the DL divided by two (DL/2). The PFAS method performance is continuously controlled and tested in a Quality Assurance/Quality Control (QA/QC) by participating in inter-laboratory comparison studies performed by Institute Nationale de Santé Publique du Québec for AMAP^68^.

#### Whole blood metal concentrations

The Institute for Bioscience-Arctic Research Centre, Aarhus University, Denmark analyzed the blood samples for 16 metals using Inductively Coupled Plasma Mass Spectrometry (ICP-MS) after digesting blood with nitric acid in the microwave. In this study, three heavy metals mercury (Hg), lead (Pb), cadmium (Cd) and the trace element selenium (Se) were included. If the value was below the DL, we assigned the value by the DL/2. The quality was ensured by repeated analyses and by frequent analysis of certified reference material (Seronorm), as well as by participation in the Quality Assurance of Information in Marine Environmental monitoring (QUASIMEME) inter-laboratory comparison program^69^.

#### Plasma cotinine concentrations

Nicotine is metabolized to cotinine in the body and the amount of cotinine in the blood is proportional to current nicotine exposure. In this study, cotinine was used as a biomarker for current smoking of tobacco. The concentration of cotinine in plasma was measured at our Centre for Arctic Health and Molecular Epidemiology (CAH-ME), Aarhus University, Denmark. The specific method was published previously^35,36^. If the value was below the DL (1 ng/ml), we assigned the value as DL/2.

#### Plasma fatty acid measurements

The traditional Greenlandic marine food has a high concentration of polyunsaturated n-3 fatty acids, whereas the modern western food has a high concentration of n-6 fatty acids. Thus, the ratio of these two fatty acids (n-3/n-6) can be used as a biomarker for traditional marine food intake relative to imported western food^70^. The fatty acids were measured at the Lipid Analytical Laboratories inc., Canada. The specific method was published previously^71^.

### Children’s health and development at follow-up

At follow-up (children aged 3–5 years), a questionnaire about the children’s health, lifestyle and development were filled out by the parents in cooperation with a health nurse at home. The questionnaires were available in Danish and Greenlandic. The questionnaires included several standardized parts, such as the “The Strength and Difficulties Questionnaire” (SDQ) and “Ages & Stages Questionnaires” (ASQ), and was developed in collaboration with the Aarhus Birth Cohort^72^ and the Greenlandic INUENDO project^52,53^, which used similar questionnaires for Danish and Greenlandic children.

From this questionnaire, data on breastfeeding duration in categories were obtained (No time, < 6 month, 6–12 months, > 12 months).

The child behavior was assessed using the SDQ being a standardized questionnaire used to assess child behavior focusing on the past six months. The SDQ has 25 assertions regarding the child, which the parents rate as “not true” (coded as 0), “somewhat true” (coded as 1) or “certainly true” (coded as 2). The 25 assertions are divided into five subscale (5 assertions each): emotional symptoms, conduct problems, hyperactivity, peer problems and prosocial behavior. From every subscale a score ranging from 0 to 10 can be calculated. A high score indicates problems, except for prosocial behavior where low scores indicate problems. The SDQ score is defined as the sum of the emotional symptoms, conduct problems, hyperactivity and peer problems scores and ranges from 0–40. In the present study, SDQ and hyperactivity score were categorized into three groups; normal (SDQ: 0–13; hyperactivity: 0–5), borderline (SDQ: 14–16; hyperactivity: 6) and abnormal (SDQ: 17–40; hyperactivity: 7–10)^73^.

### Statistical analysis

Questionnaire data were entered twice into the Epidata program version 3.1 (https://www.epidata.dk/) and exported to IBM SPSS Statistics version 26 (https://www.ibm.com/) for the statistical analysis. A significance level of 5% (*p* ≤ 0.050) were used generally and due to the low number of participants (n = 102), a level of 10% (*p* ≤ 0.100) was considered borderline significant.

The exposure variables were POPs and metals. POPs were grouped by similarities in chemical structures and properties. Afterwards, sums of chemically similar compounds were calculated (Supplementary Table S1, published previously^35^). The sums of POPs were calculated on the basis of all compounds measured within the group, but only POPs where the number of samples above the DL exceeding 50% were analyzed individually. The four single metals (Hg, Pb, Cd and Se) were analyzed regardless of the number of samples above DL.

Differences between the living locations were tested with chi-square test for categorical variables and Student’s independent T-test for continuous variables. Before the T-test, a Kolmogorov–Smirnov test, Shapiro–Wilk test and QQ-plots were used to assess the distribution. Ln-transformation improved the normality of the data, and ln-transformed data was used for the T-test.

Bivariate correlation analysis of exposures and lifestyle factors was done using Spearman’s rho.

Associations between exposure and outcome (child SDQ and hyperactivity score) were assessed with both linear and logistic regression.

The exposures were analyzed both as continuous variables and tertiles in the linear regression. The association between exposures and continuous SDQ or hyperactivity score was analyzed using multiple linear regression analysis. Linearity as assessed by partial regression plots and a plot of studentized residuals against the predicted values. Independence of residuals, as assessed by a Durbin-Watson statistic. Homoscedasticity, as assessed by visual inspection of a plot of studentized residuals versus unstandardized predicted values. Multicollinearity was assessed by tolerance values greater than 0.1 and variance inflation factor below 10. Furthermore, the assumption of normality was assessed by Q–Q plots.

Generally, the effects of the individual compounds and summed groups were evaluated without taking any possible co-exposures into consideration, however, to address the issue of potential confounding by the simultaneous exposure to other POP and metals, we performed a linear regression including multiple exposures (∑PCB, ∑OCP, ∑PFCA, ∑PFSA, Hg, Pb, Cd, and Se) as independent variables.

Odds ratios (ORs) were estimated using logistic regression analysis with continuous exposure variables and dichotomized outcome variables for both SDQ and hyperactivity score. As previously used for SDQ analysis on Greenlandic children^53^, normal and borderline were combined and compared to abnormal. Only continuous exposure variables was analyzed as unadjusted and adjusted for covariates.

The adjustments were chosen based on previous studies and a literature search^35,36^. Two main adjustment models were performed. Adjustment model 1 included maternal pregnancy data for plasma cotinine (ng/ml), education level (primary school/ high school/technical college/university) and age at delivery (year) and adjustment model 2 further included the child’s breastfeeding period (No time, < 6 month, 6–12 months, > 12 months).

Further sensitivity analyses were performed by including additional confounders in the models or stratifying the data. To explore the effect of BMI, marine intake (n-3/n-6) and GA, these factors were included as confounders in additional analyses. To investigate gender differences, the analyses with continuous exposure variable were also performed after stratifying for gender.

As the *p*-value and confidence intervals are affected by the sample size, both *p*-value and the confidence intervals (95% CI) for OR and β are given in the tables.

## Results

### Study population

#### Maternal characteristics

Baseline maternal characteristics are presented in Table 1. Of the 102 included mother–child pairs 67.7% live in Nuuk whereas the rest (32.3%) live in Sisimiut/Ilulissat. The median age at delivery was 29.2 years ranging from 20.1–41.5 years and their median pre-pregnancy BMI was 24.1 kg/m^2^ ranging from 18.7 to 43.1 kg/m^2^. No significant difference was found in age at delivery or pre-pregnancy BMI between Nuuk and Sisimiut/Ilulissat. Parity was defined as the number of prior full-term pregnancies, and women from Nuuk had significantly lower parity than women from Sisimiut/Ilulissat (both continuous and categorical parity variables (0, 1–2 and ≥ 3)). Of all the women, 20.6% were smoking during pregnancy. Smoking during pregnancy did not show any significant difference between the two living locations. As predicted the cotinine level was higher for smokers than non-smokers. The education level at pregnancy was 20.8% primary school, 12.9% secondary school, 37.6% technical school and 28.7% university. The educational level differed significantly between the locations, with women from Nuuk reporting higher educational level. Alcohol consumption before pregnancy was not significant difference between Nuuk and Sisimiut/Ilulissat, and regarding alcohol consumption during pregnancy all women either reported “ > 1 time a month” or did not answer. No significant difference in n-3/n-6 ratio was found between locations.

**Table 1:** Maternal characteristics: pregnant Greenlandic women in the ACCEPT cohort 2013–2015.

	All	Nuuk	Sisimiut and Ilulissat	*p*-Value
**Age at delivery (years), N(%)**				
	102 (100)	69 (67.7)	33 (32.3)	0.559 τ
n	102	69	33	
Mean ± SD	29.3 ± 4.56	29.4 ± 4.14	28.9 ± 5.38	
Median (min–max)	29.2 (20.1–41.5)	29.2 (21.8–38.2)	28.0 (20.1–41.5)	
Missing, n (%)	0 (0.0)	0 (0.0)	0 (0.0)	
**Pre-pregnancy BMI (kg/m**^**2**^**)**				
n	100	67	33	0.196 τ
Mean ± SD	25.6 ± 4.75	25.1 ± 4.41	26.4 ± 5.35	
Median (min–max)	24.1 (18.7–43.1)	24.0 (19.1–40.2)	24.7 (18.7–43.1)	
Missing, n (%)	2 (2.0)	2 (2.9)	0 (0.0)	
**Parity**				
n	97	67	30	0.037* **τ**
Mean ± SD	0.96 ± 1.08	0.81 ± 1.00	1.30 ± 1.18	
Median (min–max)	1 (0–4)	0 (0–4)	1 (0–4)	0.031* **χ**
0, n (%)	43 (44.3)	35 (52.2)	8 (26.7)	
1–2, n (%)	45 (46.4)	28 (41.8)	17 (56.7)	
3 or more, n (%)	9 (9.3)	4 (6.0)	5 (16.7)	
Missing, n (%)	5 (4.9)	2 (2.9)	3 (9.1)	
**Smoking during pregnancy**				
n	102	69	33	0.118 χ
No, n (%)	81 (79.4)	58 (84.1)	23 (69.7)	
Yes, (%)	21 (20.6)	11 (15.9)	10 (30.3)	
Missing, n (%)	0 (0.0)	0 (0.0)	0 (0.0)	
**P-cotinine (ng/mL)**				
n	102	69	33	
**All**				
Mean ± SD	25.9 ± 61.3	17.8 ± 47.2	42.9 ± 81.8	0.115 τ
Median (min–max)	0.50 (0.50–298)	0.50 (0.50–254)	0.50 (0.50–298)	
**Non-smokers**				
Mean ± SD	1.60 ± 20.5	1.71 ± 5.1	1.32 ± 2.8	0.729 τ
Median (min–max)	0.50 (0.50–31.7)	0.50 (0.50–31.7)	0.50 (0.50–12.1)	
**Smokers**				
Mean ± SD	119.5 ± 85.5	102.3 ± 75.3	138 ± 95.9	0.417 τ
Median (min–max)	97.8 (7.0–298)	94.2 (7.0–254)	129 (0.50–298)	
Missing, n (%)	0 (0.0)	0 (0.0)	0 (0.0)	
**Education**				
n	101	69	32	< 0.001* **χ**
Primary school, n (%)	21 (20.8)	6 (8.7)	15 (46.9)	
Secondary School, n (%)	13 (12.9)	9 (13.0)	4 (12.5)	
Technical college, n (%)	38 (37.6)	31 (44.9)	7 (21.9)	
University, n (%)	29 (28.7)	23 (33.3)	6 (18.8)	
Missing, n (%)	1 (1.0)	0 (0.0)	1 (3.0)	
**Alcohol consumption, before pregnancy**				
n	93	66	27	0.463 χ
< 1 time a month	51 (54.8)	36 (54.5)	15 (55.6)	
1 time a month	19 (20.4)	13 (19.7)	6 (22.2)	
2–3 times a month	17 (18.3)	11 (16.7)	6 (22.2)	
> 1 time a week	6 (6.5)	6 (9.1)	0 (0.0)	
Missing, n (%)	9 (8.8)	3 (4.3)	6 (18.2)	
**Alcohol consumption, during pregnancy**				
n	43	30	13	N/A
< 1 time a month	43 (100.0)	30 (100.0)	13 (100.0)	
1 time a month	0 (0.0)	0 (0.0)	0 (0.0)	
2–3 times a month	0 (0.0)	0 (0.0)	0 (0.0)	
> 1 time a week	0 (0.0)	0 (0.0)	0 (0.0)	
Missing, n (%)	59 (57.8)	39 (56.5)	20 (60.6)	
**n-3/n-6 ratio**				
n	101	68	33	0.684 τ
Mean ± SD	0.27 ± 0.11	0.27 ± 0.10	0.28 ± 0.13	
Median (min–max)	0.24 (0.10–0.73)	0.24 (0.11–0.57)	0.26 (0.10–0.73)	
Missing, n (%)	1 (1.0)	1 (1.4)	0 (0.0)	
**Lipid (g/L)**				
n	102	69	33	0.803 **τ**
Mean ± SD	6.47 ± 1.04	6.51 ± 1.11	6.44 ± 1.01	
Median (min–max)	6.50 (4.30–8.90)	6.40 (4.40–8.90)	6.50 (4.30–8.90)	
Missing, n (%)	0 (0.0)	0 (0.0)	0 (0.0)	

*N* total number of participants, *n* number of participants with information on the variable, τ: *p*-Value was calculated with student T-test on ln-transformed data, χ: *p*-Value was calculated with Chi-square test, Bold text and * = Significant (*p* ≤ 0.050), N/A: Test not applicable.

#### Child characteristics

Baseline child characteristics of the children are presented in Table 2. Of all the children, 53.9% were male and 46.1% were female. Overall, the children had median GA at birth of 40 weeks, a birth weight of 3675 g, birth length of 52 cm and head circumference of 35 cm, but all fetal growth data were significantly or borderline significantly higher in Nuuk than in Ilulissat/Sisimiut (*p* = 0.049, *p* = 0.038, *p* = 0.056, *p* = 0.055, respectively).

**Table 2:** Child characteristics: Greenlandic children born 2014–2016 in the ACCEPT birth cohort.

	All	Nuuk	Sisimiut and Ilulissat	*p*-Value
N (%)	102 (100)	69 (67.7)	33 (32.3)	
**Gender**				
Male, n (%)	55 (53.9)	40 (58.0)	15 (45.5)	0.290 χ
Female, n (%)	47 (46.1)	29 (42.0)	18 (54.5)	
Missing, n (%)	0 (0.0)	0 (0.0)	0 (0.0)	
**Gestational week at birth**				
n	102	69	33	0.049* τ
Mean ± SD	39.5 ± 1.45	39.7 ± 1.43	39.1 ± 1.42	
Median (min–max)	40 (35–42)	40 (37–42)	39 (35–41)	
Missing, n (%)	0 (0.0)	0 (0.0)	0 (0.0)	
**Birth weight (gram)**				
Mean ± SD	3671 ± 518	3743 ± 501	3521 ± 529	0.038* τ
Median (min–max)	3675 (2220–4920)	3712 (2640–4920)	3490 (2220–4505)	
Missing, n (%)	0 (0.0)	0 (0.0)	0 (0.0)	
**Birth length (cm)**				
Mean ± SD	51.6 ± 2.27	51.9 ± 2.31	51.0 ± 2.08	0.056** **τ**
Median (min–max)	52 (46–57)	52 (46–57)	51 (46–55)	
Missing, n (%)	0 (0.0)	0 (0.0)	0 (0.0)	
**Birth head circumference (cm)**				
n	101	69	32	0.055** **τ**
Mean ± SD	35.0 ± 1.51	35.1 ± 1.46	34.5 ± 1.57	
Median (min–max)	35.0 (31.0–38.5)	35.0 (32.0–38.5)	34.5 (31–38)	
Missing, n (%)	1 (1.0)	0 (0.0)	1 (3.0)	
**Breastfeeding period**				
n	101	68	33	0.085** χ
None, n (%)	4 (4.0)	2 (2.9)	2 (6.1)	
< 6 months, n (%)	17 (16.8)	8 (11.8)	9 (27.3)	
6–12 months, n (%)	31 (30.7)	20 (29.4)	11 (33.3)	
> 1 year, n (%)	49 (48.5)	38 (55.9)	11 (33.3)	
Missing, n (%)	1 (1.0)	1 (1.4)	0 (0.0)	
**Age at follow-up (months)**				
n	102	69	33	0.066** τ
Mean ± SD	53.7 ± 6.98	52.8 ± 7.20	55.4 ± 6.24	
Median (min–max)	54 (40–66)	52 (40–66)	55 (45–65)	
Missing, n (%)	0 (0.0)	0 (0.0)	0 (0.0)	
**Child behavior at follow up**				
**SDQ score**				
n	95	65	30	0.884 τ
Mean ± SD	10.2 ± 5.18	10.2 ± 4.78	10.3 ± 6.05	
Median (min–max)	9 (0–25)	9 (3–25)	9 (0–22)	
Normal, n (%)	74 (77.9)	53 (81.5)	21 (70.0)	0.247 χ
Boys/Girls, n (%)	38 (74.5)/36 (81.8)	28 (75.7)/25 (86.3)	10 (71.4)/11 (68.8)	
Borderline, n (%)	10 (10.5)	7 (10.8)	3 (10.0)	
Boys/Girls, n (%)	6 (11.8)/4 (9.1)	5 (12.5)/2 (7.1)	1 (7.1)/2 (12.5)	
Abnormal, n (%)	11 (11.6)	5 (7.7)	6 (20.0)	
Boys/Girls, n (%)	7 (13.7)/4 (9.1)	4 (10.8)/1 (3.6)	3 (21.4)/3 (18.8)	
Missing, n (%)	7 (6.9)	4 (5.8)	3 (9.1)	
**Hyperactivity score**				
N	101	69	32	0.654 τ
Mean ± SD	3.36 ± 1.89	3.57 ± 1.79	2.91 ± 2.05	
Median (min–max)	3 (0–7)	4 (0–7)	3 (0–7)	
Normal, n (%)	88 (87.1)	60 (87.0)	28 (87.5)	0.899 χ
Boys/Girls, n (%)	46 (83.6)/42 (91.3)	33 (82.5)/27 (93.1)	13 (86.7)/15 (88.2)	
Borderline, n (%)	8 (7.9)	5 (7.2)	3 (9.4)	
Boys/Girls, n (%)	5 (9.1)/3 (6.5)	3 (7.5)/2 (6.9)	2 (13.3)/1 (5.9)	
Abnormal, n (%)	5 (5.0)	4 (5.8)	1 (3.1)	
Boys/Girls, n (%)	4 (7.3)/1 (2.2)	4 (10.0)/0 (0.0)	0 (0.0)/1 (5.9)	
Missing, n (%)	1 (1.0)	0 (0.0)	1 (3.0)	

*N* total number of participants, *n* number of participants with information on the variable, τ: *p*-Value was calculated with T-test on ln-transformed data, χ: *p*-Value was calculated with Chi-square test for locations, Bold text and * = Significant (*p* ≤ 0.050), Bold text and ** = Borderline significant (*p* ≤ 0.100), Missing % was calculated on the basis of all responders and non-responders.

Children in Nuuk were also breastfed slightly longer than in Sisimiut/Ilulissat, thus a borderline significant difference was found (*p* = 0.085).

The median age of the children at follow-up was 54 months (4.5 years) ranging from 40 months (3.3 years) to 66 months (5.5 years). The age at follow-up was borderline significantly different between the locations (Nuuk: 52 months, Sisimiut/Ilulissat: 55 months; *p* = 0.066).

The median SDQ score was 9 ranging from 0 to 25, and 77.9% had a normal, 10.5% borderline and 11.6% abnormal SDQ score. A higher proportion of boys (13.7%) than girls (9.1%) had an abnormal SDQ score. The median hyperactivity score was 3 ranging from 0 to 7, and 87.1% had a normal, 7.9% borderline and 5.0% abnormal hyperactivity score. A higher proportion of boys (7.3%) than girls (2.2%) had an abnormal hyperactivity score (Table 2).

### Serum concentrations of POPs and metals

Maternal exposure to POPs, sums of POPs and metals stratified between Nuuk and Sisimiut/Ilulissat is presented in Table 3. The concentration of almost all individual PCBs and ∑PCBs showed a significant difference between Nuuk and Sisimiut/Ilulissat being highest in Sisimiut/Ilulissat (*p* = 0.008–0.040). The difference in PCB170 was borderline significant (*p* = 0.084) and PCB156 showed no significant location difference. The concentrations of OCPs showed a similar significant different pattern between the two locations (*p* = 0.001–0.008; except ß-HCH *p* = 0.071). Thus, a significant difference between the two locations was observed for both ∑LegacyPOPs and ∑LipophilicPOPs.

**Table 3:** Concentrations of POPs and metals: Greenlandic pregnant women in the ACCEPT cohort 2013–2015.

	% over DL	All (N = 102)		Nuuk (N = 69)		Sisimiut and Ilulissat (N = 33)		*p*-Value
		GM ± GSD	Median (min–max)	GM ± GSD	Median (min–max)	GM ± GSD	Median (min–max)	
**PCBs (µg/kg lipid)**								
PCB118	98	7.91 ± 2.15	8.65 (0.50–47.0)	6.97 ± 2.19	7.30 (0.50–32.0)	10.3 ± 1.95	9.90 (2.30–47.0)	0.016*
PCB138	100	22.3 ± 2.04	23.0 (2.40–110)	19.8 ± 2.06	20.0 (2.40–93.0)	28.7 ± 1.87	29.0 (7.70–110)	0.013*
PCB153	100	46.7 ± 2.09	48.0 (5.10–250)	40.9 ± 2.09	42.0 (5.10–220)	61.8 ± 1.96	60.0 (16.0–250)	0.008*
PCB156	75.5	2.55 ± 2.34	2.70 (0.50–16.0)	2.40 ± 2.35	2.70 (0.50–16.0)	2.89 ± 2.32	2.80 (0.50–13.0)	0.308
PCB170	99	7.72 ± 2.04	7.85 (1.30–39.0)	7.10 ± 2.06	7.40 (1.30–39.0)	9.21 ± 1.95	8.40 (2.80–35.0)	0.084**
PCB180	100	23.0 ± 2.08	22.5 (3.80–120)	20.7 ± 2.08	22.0 (3.80–120)	28.5 ± 2.02	27.0 (8.70–110)	0.040*
PCB183	76.5	2.48 ± 2.22	2.75 (0.50–12.0)	2.16 ± 2.23	2.30 (0.50–11.0)	3.33 ± 2.04	3.60 (0.50–12.0)	0.009*
PCB187	97	10.6 ± 2.05	11.0 (1.60–48.0)	9.34 ± 2.03	9.00 (1.60–48.0)	13.8 ± 1.95	12.0 (3.60–44.0)	0.010*
PCB99	66.7	6.38 ± 2.24	7.00 (1.50–39.0)	5.54 ± 2.22	6.00 (1.50–32.0)	8.57 ± 2.14	10.0 (2.00–39.0)	0.010*
**∑PCBs**		159 ± 1.88	167 (37.7–675)	142 ± 1.87	144 (37.7–648)	200 ± 1.79	187 (66.6–675)	0.010*
**OCPs (µg/kg lipid)**								
cis-Nonachlor	97	6.91 ± 2.20	7.35 (0.70–32.0)	5.95 ± 2.22	6.40 (0.70–26.0)	9.46 ± 1.99	10.0 (1.20–32.0)	0.005*
Hexachlorobenzene	99	26.0 ± 1.87	27.0 (2.50–110)	23.0 ± 1.84	24.0 (2.50–71.0)	33.7 ± 1.81	32.0 (10.0–110)	0.004*
Mirex	63.7	2.15 ± 2.45	2.50 (0.50–12.0)	1.76 ± 2.34	2.00 (0.50–9.70)	3.23 ± 2.37	3.00 (0.50–12.0)	0.001*
Oxychlordane	98	15.6 ± 2.35	16.5 (1.00–77.0)	13.0 ± 2.36	15.0 (1.00–77.0)	22.7 ± 2.07	24.0 (4.40–76.0)	0.002*
p,p’-DDE	99	108 ± 2.16	110 (5.00–530)	93.6 ± 2.17	93.0 (5.00–350)	144 ± 1.96	160 (31.0–530)	0.008*
ß-HCH	86	3.29 ± 2.03	3.50 (0.50–16.0)	3.02 ± 2.07	3.40 (0.50–9.40)	3.96 ± 1.89	3.90 (0.50–16.0)	0.071**
trans-Nonachlor	96	36.2 ± 2.29	39.5 (3.10–180)	30.9 ± 2.31	35.0 (3.10–150)	50.4 ± 2.04	49.0 (7.60–180)	0.005*
**∑OCPs**		209 ± 2.04	213 (17.6–939)	182 ± 2.04	174 (17.6–637)	278 ± 1.90	293 (64.6–939)	0.005*
**∑LegacyPOPs**		371 ± 1.93	372 (55.3–1613)	328 ± 1.92	322 (55.3–1216)	482 ± 1.82	484 (149–1613)	0.005*
**∑LipophilicPOPs**		392 ± 1.85	389 (70.3–1642)	345 ± 1.85	332 (70.3–1245)	505 ± 1.78	488 (168–1642)	0.004*
**PFSAs (ng/mL)**								
PFHxS	99	0.39 ± 1.70	0.38 (0.04–1.07)	0.36 ± 1.77	0.37 (0.04–1.07)	0.44 ± 1.50	0.44 (0.19–0.88)	0.081**
PFHpS	74	0.13 ± 1.85	0.14 (0.06–0.36)	0.12 ± 1.88	0.14 (0.06–0.36)	0.17 ± 1.62	0.17 (0.06–0.36)	0.001*
PFOS	100	7.94 ± 1.64	8.44 (1.45–19.2)	7.33 ± 1.65	7.64 (1.45–19.0)	9.36 ± 1.57	10.3 (3.86–19.2)	0.019*
**∑PFSAs**		9.39 ± 1.55	9.83 (2.40–21.1)	8.76 ± 1.55	8.95 (2.40–20.8)	10.9 ± 1.51	11.7 (5.04–21.1)	0.019*
**PFCAs (ng/mL)**								
PFOA	99	0.95 ± 1.88	0.99 (0.10–7.26)	0.99 ± 1.93	0.99 (0.18–7.26)	0.86 ± 1.77	0.98 (0.10–1.85)	0.270
PFNA	100	1.07 ± 1.59	1.07 (0.21–3.26)	0.99 ± 1.61	1.01 (0.21–3.26)	1.25 ± 1.49	1.18 (0.59–2.87)	0.019*
PFDA	100	0.71 ± 1.77	0.71 (0.14–2.32)	0.67 ± 1.76	0.68 (0.14–2.32)	0.83 ± 1.75	0.87 (0.20–2.08)	0.075**
PFUnA	99	1.31 ± 2.38	1.47 (0.15–8.12)	1.23 ± 2.32	1.38 (0.15–8.12)	1.50 ± 2.51	1.79 (0.17–6.19)	0.292
**∑PFCAs**		5.73 ± 1.64	5.53 (1.43–17.1)	5.54 ± 1.67	5.41 (1.43–17.1)	6.13 ± 1.58	5.59 (2.52–16.7)	0.338
**∑PFASs (ng/mL)**								
∑PFASs		15.4 ± 1.54	15.6 (3.83–35.8)	14.6 ± 1.55	15.2 (3.83–35.8)	17.2 ± 1.49	17.6 (8.64–35.7)	0.067**
**Metals (µg/L)**								
Hg	66	3.79 ± 2.17	4.06 (0.42–69.3)	3.30 ± 1.82	3.47 (0.58–17.9)	5.05 ± 2.71	5.25 (0.42–69.3)	0.029*
Pb	98	7.27 ± 1.75	6.55 (1.58–64.1)	7.03 ± 1.85	6.34 (1.58–64.1)	7.80 ± 1.53	8.40 (3.15–27.3)	0.381
Cd	43.1	0.58 ± 2.26	0.53 (0.11–3.15)	0.52 ± 2.38	0.53 (0.11–3.15)	0.73 ± 1.92	0.53 (0.21–3.15)	0.052**
Se	100	141 ± 1.57	130 (65.4–1065)	128 ± 1.37	117 (65.4–292)	173 ± 1.83	156 (79.8–1065)	0.009*

*N* number of participants, *GM* geometric mean, *GSD* geometric standard deviation, *DL* detection limit, *p*-Values was calculated with T-test on ln-transformed data. Bold text and * = Significant (*p* ≤ 0.050), Bold text and ** = Borderline significant (*p* ≤ 0.100).

Significantly higher concentration of two PFSAs; PFHpS and PFOS were observed in Sisimiut/Ilulissat (*p* = 0.001 and *p* = 0.019, respectively). Whereas the concentration of PFHxS was borderline significantly higher in Sisimiut/Ilulissat (*p* = 0.081). Likewise, two PFCAs, PFNA and PFDA, were significantly or borderline significantly higher in Sisimiut/Ilulissat compared to Nuuk (*p* = 0.019 and *p* = 0.075, respectively). Moreover, the sum of PFSAs and PFASs was significantly higher in Sisimiut/Ilulissat compared to Nuuk (*p* = 0.019 and *p* = 0.067, respectively).

The metal concentrations, except for Pb, also varied significantly or borderline significantly between the two locations being highest in Sisimiut/Ilulissat (Hg: *p* = 0.029, Se: *p* = 0.009, Cd: *p* = 0.052). As shown in Supplementary Table S2, serum lipPOP, PFAS and whole blood levels of Hg, Pb, Cd and Se correlated positively.

### Correlations between serum POPs, metals and lifestyle factors

The Spearman correlation coefficients for the correlations between sums of POPs or metals and maternal lifestyle factors (age, BMI, cotinine concentration and n-3/n-6 ratio) are presented in Supplementary Table S3. The ∑PCBs, ∑OCPs, ∑LegacyPOPs and ∑LipophilicPOPs showed a significant positive correlation with age (r_s_ = 0.213–0.262, *p* = 0.008–0.036), whereas in contrast these POP groups were inverse correlated with BMI (r_s_ = − 0.238 - − 0.193, *p* = 0.020–0.054). The ∑OCPs positively and significantly correlated with cotinine (r_s_ = 0.197, *p* = 0.048) and borderline significant with n-3/n-6 (r_s_ = 0.183, *p* = 0.067). Borderline significant positive correlation was found between the ∑LegacyPOPs and cotinine (r_s_ = 0.172, *p* = 0.084).

The ∑PFSAs, ∑PFCAs and ∑PFASs were positively significant correlated with n-3/n-6 ratio (r_s_ = 0.252–0.293, *p* = 0.003–0.011), but not significantly correlated with any of the other lifestyle variables. For the metals, Pb and Cd had a significant positive correlation with cotinine, r_s_ = 0.292, *p* = 0.003 and r_s=_ 0.544, *p* < 0.001, respectively. Se was significantly positive correlated with n-3/n-6 ratio (r_s_ = 0.255, *p* = 0.010). Finally, Cd showed a borderline significant inverse correlation with n-3/n-6 ratio (r_s_ = − 0.182, *p* = 0.068) (Supplementary Table S3).

### Associations between prenatal POP and metal exposure and continuous SDQ score

Table 4 shows the linear regression analysis of the associations between prenatal OCP exposure and continuous SDQ score. Two adjustment models were performed: Model 1 was adjusted for maternal plasma cotinine, educational level, and age at delivery; Model 2 was further adjusted for breast-feeding duration. None of the continuous OCP exposure variables were significant associated with SDQ score (Table 4). For the categorical exposure variables, the medium tertile of Hexachlorobenezene was significantly positively associated with continuous SDQ score in both adjustment models (Model 1 β = 3.06, *p* = 0.010, Model 2 β = 2.95, *p* = 0.014), but the association was non-significant in the highest tertile. The medium ß-HCH tertile showed a borderline significant positive association with continuous SDQ score in the unadjusted data (β = 2.20, *p* = 0.083), and significantly after adjustments (Model 1 β = 3.58, *p* = 0.004, Model 2 β = 3.51, *p* = 0.005), but the highest tertile was only borderline significant in the adjustment model 1 (β = 2.03, *p* = 0.100). Trans-nonachlor borderline positively associated with SDQ score in in the medium tertile in the adjustment model 1 (β = 2.06, *p* = 0.082) (Table 4).

**Table 4:** Linear regression analysis of associations between prenatal OCP exposure and continuous SDQ score: Greenlandic children 3–5 years of age born 2014–2016 in the ACCEPT birth cohort.

All (n = 95)						
OCPs (µg/kg lipid)	Unadjusted		Adjustment model 1		Adjustment model 2	
	β (95% CI)	*p*-Value	β (95% CI)	*p*-Value	β (95% CI)	*p*-Value
**cis-Nonachlor**						
Cont	0.05 (− 0.11, 0.21)	0.533	0.07 (− 0.10, 0.24)	0.403	0.06 (− 0.11, 0.24)	0.457
Low	Ref		Ref		Ref	
Med	0.05 (− 2.45, 2.56)	0.967	1.38 (− 1.01, 3.77)	0.258	1.17 (− 1.29, 3.63)	0.351
High	0.18 (− 2.75, 2.38)	0.889	1.36 (− 1.13, 3.84)	0.286	1.22 (− 1.32, 3.76)	0.346
p-trend		0.892		0.795		0.938
**Hexachlorobenzene**						
Cont	0.02 (− 0.04, 0.07)	0.543	0.02 (− 0.04, 0.07)	0.566	0.01 (− 0.04, 0.07)	0.641
Low	Ref		Ref		Ref	
Med	1.91 (− 0.59, 4.40)	0.134	3.06 (0.74, 5.39)	0.010*	2.95 (0.60, 5.30)	0.014*
High	0.31 (− 2.21, 2.82)	0.812	1.71 (− 0.68, 4.10)	0.161	1.59 (− 0.83, 4.01)	0.197
p-trend		0.805		0.555		0.668
**Mirex**						
Cont	− 0.11 (− 0.51, 0.29)	0.572	− 0.12 (− 0.53, 0.29)	0.557	− 0.14 (− 0.55, 0.27)	0.503
Low	Ref		Ref		Ref	
Med	− 1.88 (− 4.34, 0.58)	0.134	− 0.66 (− 3.16, 1.85)	0.608	− 0.97 (− 3.55, 1.61)	0.462
High	− 1.14 (− 3.64, 1.37)	0.374	0.17 (− 2.31, 2.65)	0.892	− 0.01 (− 2.49, 2.46)	0.991
p-trend		0.351		0.444		0.385
**Oxychlordane**						
Cont	0.03 (− 0.03, 0.09)	0.404	0.03 (− 0.04, 0.09)	0.415	0.02 (− 0.04, 0.08)	0.475
Low	Ref		Ref		Ref	
Med	− 0.52 (− 3.00, 1.97)	0.684	0.78 (− 1.64, 3.20)	0.528	0.56 (− 1.89, 3.02)	0.654
High	0.28 (− 2.28, 2.85)	0.829	1.57 (− 0.88, 4.01)	0.209	1.42 (− 1.07, 3.91)	0.262
p-trend		0.849		0.630		0.757
**p,p’-DDE**						
Cont	0.01 (− 0.01, 0.02)	0.330	0.01 (− 0.01, 0.02)	0.322	0.01 (− 0.01, 0.02)	0.368
Low	Ref		Ref		Ref	
Med	− 0.06 (− 2.55, 2.43)	0.962	0.42 (− 2.03, 2.86)	0.738	0.22 (− 2.29, 2.72)	0.866
High	0.66 (− 1.95, 3.26)	0.621	1.93 (− 0.58, 4.45)	0.132	1.74 (− 0.81, 4.28)	0.180
p-trend		0.631		0.422		0.519
**ß-HCH**						
Cont	0.18 (− 0.22, 0.58)	0.371	0.26 (− 0.17, 0.68)	0.231	0.24 (− 0.19, 0.67)	0.264
Low	Ref		Ref		Ref	
Med	2.20 (− 0.29, 4.68)	0.083**	3.58 (1.16, 6.00)	0.004*	3.51 (1.04, 5.98)	0.005*
High	0.48 (− 2.04, 3.01)	0.707	2.03 (− 0.39, 4.45)	0.100**	1.95 (− 0.53, 4.43)	0.123
p-trend		0.715		0.419		0.523
**trans-Nonachlor**						
Cont	0.01 (− 0.02, 0.04)	0.418	0.02 (− 0.01, 0.04)	0.319	0.01 (− 0.02, 0.04)	0.366
Low	Ref		Ref		Ref	
Med	0.68 (− 1.79, 3.14)	0.590	2.06 (− 0.26, 4.39)	0.082**	1.90 (− 0.48, 4.27)	0.117
High	− 0.11 (− 2.70, 2.48)	0.935	1.61 (− 0.91, 4.14)	0.210	1.52 (− 1.01, 4.09)	0.249
p-trend		0.962		0.660		0.788
**∑OCPs**						
Cont	0.00 (− 0.00, 0.01)	0.377	0.00 (− 0.00, 0.01)	0.349	0.00 (− 0.00, 0.01)	0.400
Low	Ref		Ref		Ref	
Med	0.97 (− 1.51, 3.45)	0.443	1.97 (− 0.46, 4.41)	0.113	1.83 (− 0.65, 4.31)	0.149
High	0.18 (− 2.38, 2.74)	0.889	1.36 (− 1.06, 3.78)	0.270	1.22 (− 1.26, 3.69)	0.335
p-trend		0.868		0.650		0.780

*n* Number of participants in parameter, *β* Linear regression coefficient in score points, *CI* confidence interval, Bold text and * = Significant (*p* ≤ 0.050), Bold text and ** = Borderline significant (*p* ≤ 0.100), Adjustment model 1: Maternal plasma cotinine, maternal educational level, maternal age at delivery, Adjustment model 2: Adjustment model 1 + breast-feeding duration, Med = Medium, Cont. = Continuous, *OCPs* organochlorine pesticides.

Supplementary Table S4 shows the linear regression analysis of the associations between prenatal PCB, PFAS and metal exposure and continuous SDQ score. None of the continuous PCB or PFAS exposure variables were significant associated with SDQ score. However, the continuous Hg exposure variables was significant negatively associated with SDQ scores (unadjusted β = − 0.11, *p* = 0.093, Model 1 β = − 0.12, *p* = 0.066, Model 2 β = − 0.14, *p* = 0.040) and continuous Se was weakly negative associated with SDQ scores (unadjusted β = − 0.01, *p* = 0.050, Model 1 β = − 0.01, *p* = 0.044, Model 2 β = − 0.01, *p* = 0.028) (Supplementary Table S4).

In the analyses with categorical PCB exposure variables, some significant or borderline significant associations with SDQ scores were found. The PCB170 showed inverse association with the unadjusted continuous SDQ score in the medium tertile (β = − 2.88, *p* = 0.019) and highest tertile (β = − 2.18, *p* = 0.089). However, the associations were non-significant in the adjusted models (Supplementary Table S4). We found also for the unadjusted analysis for ∑PCB a borderline significant association with SDQ score in the medium tertile (β = − 2.23, *p* = 0.074), but the significance disappeared upon adjustment (Supplementary Table S4). No significant associations were found for the categorical metal or PFAS and SDQ score (Supplementary Table S4).

When multiple exposures (∑PCB, ∑OCP, ∑PFCA, ∑PFSA, Hg, Pb, Cd, and Se) were included in one linear regression model, we found that ∑OCP was significantly positive associated with SDQ scores (unadjusted β = 0.02, *p* = 0.009, Model 1 β = 0.02, *p* = 0.010, Model 2 β = 0.02, *p* = 0.007), while ∑PCB was borderline significantly inverse associated (unadjusted β = − 0.02, *p* = 0.061, Model 1 β = − 0.02, *p* = 0.075, Model 2 β = − 0.02, *p* = 0.074) (Supplementary Table S5).

In the analyses with continuous exposure variables stratified by gender (Supplementary Table S6), the estimates were generally similar to those in the pooled analyses. For boys, Se was inversely borderline significant associated with SDQ scores (unadjusted β = − 0.01, *p* = 0.075, Model 1 β = − 0.01, *p* = 0.069). Similar, but non-significant results were seen for girls (unadjusted β = − 0.01, *p* = 0.553, Model 1 β = − 0.01, *p* = 0.632).

### Associations between prenatal POP and metal exposure and continuous hyperactivity score

Table 5 shows the linear regression analyses of the associations between prenatal OCP exposure and continuous hyperactivity score. No significant or borderline significant associations between continuous OCP exposure variables and continuous hyperactivity score were found. In the analyses with categorical OCP exposure variables, the medium tertile of Mirex was significantly inversely associated with continuous hyperactivity score (Model 1 β = − 1.18, *p* = 0.010, Model 2 β = − 1.35, *p* = 0.004), whereas non-significant in the highest tertile (Table 5).

**Table 5:** Linear regression analysis of the associations between prenatal OCP exposure and continuous hyperactivity score: Greenlandic children 3–5 years of age born 2014–2016, the ACCEPT birth cohort.

All (n = 101)						
OCPs (µg/kg lipid)	Unadjusted		Adjustment model 1		Adjustment model 2	
	β (95% CI)	*p*-Value	β (95% CI)	*p*-Value	β (95% CI)	*p*-Value
**cis-Nonachlor**						
Cont	0.03 (− 0.03, 0.09)	0.279	0.03 (− 0.03, 0.09)	0.311	0.03 (− 0.03, 0.10)	0.278
Low	Ref		Ref		Ref	
Med	− 0.49 (− 1.38, 0.41)	0.287	− 0.44 (− 1.33, 0.46)	0.341	− 0.40 (− 1.33, 0.53)	0.398
High	0.01 (– 0.88, 0.91)	0.975	0.19 (– 0.73, 1.10)	0.686	0.22 (– 0.72, 1.15)	0.651
p-trend		0.967		0.967		0.884
**Hexachlorobenzene**						
Cont	0.00 (− 0.02, 0.02)	0.797	0.00 (− 0.02, 0.02)	0.873	0.00 (− 0.02, 0.02)	0.814
Low	Ref		Ref		Ref	
Med	− 0.20 (− 1.09, 0.70)	0.665	− 0.24 (− 1.13, 0.65)	0.603	− 0.22 (− 1.13, 0.68)	0.627
High	− 0.09 (− 1.00, 0.82)	0.844	0.08 (− 0.85, 1.00)	0.874	0.09 (− 0.85, 1.04)	0.848
p-trend		0.846		0.822		0.887
**Mirex**						
Cont	− 0.01 (− 0.15, 0.13)	0.882	− 0.02 (− 0.17, 0.13)	0.807	− 0.02 (− 0.16, 0.13)	0.843
Low	Ref		Ref		Ref	
Med	− 1.01 (− 1.87, − 0.14)	0.023*	− 1.18 (− 2.08, − 0.28)	0.010*	− 1.35 (− 2.23, − 0.43)	0.004*
High	− 0.26 (− 1.12, 0.61)	0.561	− 0.14 (− 1.02, 0.75)	0.766	− 0.23 (− 1.12, 0.66)	0.616
p-trend		0.517		0.447		0.467
**Oxychlordane**						
Cont	0.01 (− 0.01, 0.03)	0.234	0.01 (− 0.01, 0.04)	0.271	0.01 (− 0.01, 0.04)	0.246
Low	Ref		Ref		Ref	
Med	− 0.56 (− 1.44, 0.32)	0.215	− 0.71, (− 1.60, 0.19)	0.121	− 0.71 (− 1.62, 0.21)	0.129
High	0.14 (− 0.75, 1.03)	0.764	0.23 (− 0.66, 1.11)	0.617	0.25 (− 0.65, 1.16)	0.585
p-trend		0.910		0.973		0.946
**p,p’-DDE**						
Cont	0.00 (− 0.00, 0.01)	0.237	0.00 (− 0.00. 0.01)	0.284	0.00 (− 0.00, 0.01)	0.256
Low	Ref		Ref		Ref	
Med	− 0.32 (− 1.20, 0.56)	0.476	− 0.59 (− 1.50, 0.32)	0.205	− 0.59 (− 1.53, 0.35)	0.217
High	0.06 (− 0.86, 0.98)	0.895	0.10 (− 0.83, 1.04)	0.830	0.13 (− 0.82, 1.08)	0.788
p-trend		0.780		0.853		0.776
**ß-HCH**						
Cont	0.08 (− 0.06, 0.22)	0.269	0.08 (− 0.07, 0.23)	0.294	0.09 (− 0.07, 0.25)	0.260
Low	Ref		Ref		Ref	
Med	0.50 (− 0.39, 1.39)	0.270	0.55 (− 0.37, 1.48)	0.240	0.65 (− 0.29, 1.82)	0.177
High	0.22 (− 0.68, 1.11)	0.637	0.46 (− 0.48, 1.39)	0.337	0.53 (− 0.43, 1.49)	0.278
p-trend		0.637		0.650		0.566
**trans-Nonachlor**						
Cont	0.01 (− 0.00, 0.02)	0.208	0.01 (− 0.00, 0.02)	0.231	0.01 (− 0.00, 0.02)	0.209
Low	Ref		Ref		Ref	
Med	− 0.50 (− 1.38, 0.38)	0.265	− 0.48 (− 1.35, 0.40)	0.285	− 0.44 (− 1.34, 0.45)	0.334
High	0.19 (− 0.71, 1.08)	0.685	0.35 (− 0.58, 1.28)	0.458	0.39 (− 0.55, 1.34)	0.416
p-trend		0.712		0.725		0.649
**∑OCPs**						
Cont	0.00 (− 0.00, 0.00)	0.265	0.00 (− 0.00, 0.00)	0.309	0.00 (− 0.00, 0.00)	0.278
Low	Ref		Ref		Ref	
Med	− 0.32 (− 1.21, 0.56)	0.473	− 0.56 (− 1.47, 0.34)	0.223	− 0.52 (− 1.45, 0.41)	0.271
High	0.34 (− 0.55, 1.24)	0.449	0.37 (− 0.52, 1.26)	0.415	0.40 (− 0.51, 1.31)	0.391
p-trend		0.466		0.526		0.457

*n* Number of participants in parameter, *β* Linear regression coefficient in score points, *CI* confidence interval, Bold text and *= Significant (*p* ≤ 0.050), Bold text and ** = Borderline significant (*p* ≤ 0.100), Adjustment model 1: Maternal plasma cotinine, maternal educational level, maternal age at delivery, Adjustment model 2: Adjustment model 1 + breast-feeding duration, Med = Medium, Cont. = Continuous, *OCPs* organochlorine pesticides.

Supplementary Table S7 shows the linear regression analyses of the associations between prenatal PCB, PFAS and metal exposure and continuous hyperactivity score. No significant or borderline significant associations between continuous PCB, PFAS and metal exposure variables and continuous hyperactivity score were found. In the analyses with categorical exposure variables, the medium tertile of ∑PFCAs was significantly inversely associated with continuous hyperactivity score (unadjusted β = − 1.15, *p* = 0.009, Model 1 β = − 1.07, *p* = 0.015, Model 2 β = − 1.14, *p* = 0.012), but not significant for the highest tertile (Supplementary Table S7).

When multiple exposures (∑PCB, ∑OCP, ∑PFCA, ∑PFSA, Hg, Pb, Cd, and Se) were included in one linear regression model, no significant associations between the continuous exposures and hyperactivity scores were found (Supplementary Table S8).

In the analyses with continuous exposure variables stratified by gender (Supplementary Table S9), the estimates were generally similar to those in the pooled analyses. Only one borderline significant result was seen, Se was inversely associated with hyperactivity scores among boys in the unadjusted analyses (β = − 0.00, *p* = 0.087).

### Associations between prenatal POP and metal exposure and risk of abnormal SDQ score

Table 6 shows the logistic regression analyses of the associations between prenatal OCP exposure and the risk of abnormal SDQ score. Of the children, 11.6% (11/95) had an abnormal SDQ score.

**Table 6:** Logistic regression analysis of associations between continuous prenatal OCP exposure and abnormal SDQ score: Greenlandic children 3–5 years of age born 2014–2016, the ACCEPT birth cohort.

	Unadjusted		Adjustment model 1		Adjustment model 2	
	OR (95% CI)	*p*-Value	OR (95% CI)	*p*-Value	OR (95% CI)	*p*-Value
**OCPs (µg/kg lipid)**						
cis-Nonachlor	1.08 (1.00, 1.18)	0.058**	1.09 (0.99, 1.20)	0.079**	1.09 (0.99, 1.20)	0.099**
Hexachlorobenzene	1.02 (1.00, 1.05)	0.091**	1.02 (0.99, 1.05)	0.237	1.02 (0.99, 1.05)	0.295
Mirex	1.13 (0.92, 1.39)	0.258	1.08 (0.86, 1.36)	0.488	1.07 (0.85, 1.35)	0.553
Oxychlordane	1.02 (0.99, 1.06)	0.106	1.02 (0.99, 1.05)	0.216	1.02 (0.99, 1.05)	0.264
p,p’-DDE	1.01 (1.00, 1.01)	0.031*	1.01 (1.00, 1.01)	0.077**	1.01 (1.00, 1.01)	0.092**
ß-HCH	1.15 (0.95, 1.40)	0.150	1.15 (0.91, 1.44)	0.248	1.13 (0.89,1.44)	0.301
trans-Nonachlor	1.01 (1.00, 1.03)	0.063**	1.01 (1.00, 1.03)	0.091**	1.01 (1.00, 1.03)	0.113
**∑OCPs**	1.00 (1.00, 1.01)	0.041*	1.00 (1.00, 1.01)	0.094**	1.00 (1.00, 1.01)	0.114

Normal/borderline n = 84, Abnormal n = 11, *OR* odds-ratio, *CI* confidence interval, Bold text and * = Significant (*p* ≤ 0.050), Bold text and ** = Borderline significant (*p* ≤ 0.100), Adjustment model 1: Maternal plasma cotinine, maternal educational level, maternal age at delivery, Adjustment model 2: Adjustment model 1 + breast-feeding duration, *OCPs* Organochlorine pesticides.

Several OCPs were associated with the risk of abnormal SDQ. The continuous cis-Nonachlor variable was borderline significant positively associated with abnormal SDQ-score, both in the unadjusted (OR = 1.08, *p* = 0.058) as well as all the adjusted (Model 1 OR = 1.09, *p* = 0.079; Model 2 OR = 1.09, *p* = 0.099) analyses. A borderline significant association was found between continuous hexachlorobenzene and abnormal SDQ-score (unadjusted OR = 1.02, *p* = 0.091), however the significance disappeared upon adjustment. In addition, the continuous p,p’DDE data was significantly or borderline associated with abnormal SDQ-score (unadjusted OR = 1.01, *p* = 0.031; Model 1 OR = 1.01, *p* = 0.077; Model 2 OR = 1.01, *p* = 0.092). Significant or borderline significant positive associations were found for continuous trans-Nonachlor (unadjusted OR = 1.01, *p* = 0.063) and ∑OCPs (unadjusted OR = 1.00, *p* = 0.041). The associations were also borderline significant in the adjustment model 1 (trans-Nonachlor OR = 1.01, *p* = 0.091; ∑OCPs OR = 1.00, *p* = 0.094), but were further weakened in adjustment model 2 (including breastfeeding period). When comparing the adjustment model 1 to adjustment model 2, breastfeeding seems to weaken the association between the OCP variables and abnormal SDQ score (Table 6).

Supplementary Table S10 shows the logistic regression analyses of the associations between prenatal PCB, PFAS and metal exposure and the risk of abnormal SDQ score. None of the PCBs, PFASs or metals were associated with abnormal SDQ score (Supplementary Table S10).

In the analyses with continuous exposure variables stratified by gender, the statistical power was markedly reduced due to the low number of children with abnormal SDQ scores (boys n = 7, girls n = 4) Supplementary Table S11). However, several OCPs were significantly or borderline significantly associated with increased risk of abnormal SDQ scores among the boys in the unadjusted model: cis-Nonachlor (OR = 1.10, *p* = 0.073), Mirex (OR = 1.25, *p* = 0.091), p,p’-DDE (OR = 1.01, *p* = 0.033), trans-Nonachlor (OR = 1.02, *p* = 0.085) and the ∑OCP (OR = 1.00, *p* = 0.047). For p,p’-DDE, the results were borderline significant in the adjusted model 1 (OR = 1.01, *p* = 0.074). Among the girls, the associations were non-significant and generally weaker. The only borderline significant association found for girls was for PFOA in the adjusted model 1 (OR = 0.03, *p* = 0.060) (Supplementary Table S11).

### Associations between prenatal POP and metal exposure and risk of abnormal hyperactivity score

Table 7 shows the logistic regression analysis of the associations between prenatal OCP exposure and the risk of abnormal hyperactivity score. Only 5.0% (5/101) of the children had an abnormal hyperactivity score.

**Table 7:** Logistic regression analysis of associations between prenatal OCP exposure and abnormal hyperactivity score: Greenlandic children 3–5 years of age born 2014–2016, the ACCEPT birth cohort.

	Unadjusted		Adjustment model 1		Adjustment model 2	
	OR (95% CI)	*p*-Value	OR (95% CI)	*p*-Value	OR (95% CI)	*p*-Value
**OCPs (µg/kg lipid)**						
cis-Nonachlor	1.07 (0.96, 1.20)	0.240	1.10 (0.97, 1.24)	0.148	1.09 (0.97, 1.24)	0.151
Hexachlorobenzene	1.02 (0.98, 1.05)	0.417	1.02 (0.98, 1.06)	0.276	1.02 (0.98, 1.06)	0.289
Mirex	1.21 (0.93, 1.58)	0.164	1.28 (0.96, 1.72)	0.096**	1.28 (0.96, 1.72)	0.099**
Oxychlordane	1.03 (0.99, 1.07)	0.105	1.04 (1.00, 1.08)	0.066**	1.04 (1.00, 1.08)	0.074**
p,p’-DDE	1.00 (1.00, 1.01)	0.469	1.00 (1.00, 1.01)	0.342	1.00 (1.00, 1.01)	0.351
ß-HCH	1.12 (0.86, 1.46)	0.400	1.20 (0.88, 1.64)	0.245	1.19 (0.88, 1.61)	0.254
trans-Nonachlor	1.02 (1.00, 1.03)	0.118	1.02 (1.00, 1.04)	0.071**	1.02 (1.00, 1.04)	0.077**
**∑OCPs**	1.00 (1.00, 1.01)	0.290	1.00 (1.00, 1.01)	0.189	1.00 (1.00, 1.01)	0.200

Normal/borderline n = 96, Abnormal n = 5, *OR* odds-ratio, *CI* confidence interval, Bold text and * = Significant (*p* ≤ 0.050), Bold text and ** = Borderline significant (*p* ≤ 0.100), Adjustment model 1: Maternal plasma cotinine, maternal educational level, maternal age at delivery, Adjustment model 2: Adjustment model 1 + breast-feeding duration, *OCPs* organochlorine pesticides.

In the analyses with continuous exposure variables, several OCP were associated with an increased risk of abnormal hyperactivity score. All associations were non-significant in the unadjusted analyses, but borderline significant after adjustment; Mirex (Model 1 OR = 1.28, *p* = 0.096), Oxychlordane (Model 1 OR = 1.04, *p* = 0.066) and trans-Nonachlor (Model 1 OR = 1.02, *p* = 0.071). The association were similar in adjustment model 2, being further adjusted for the breastfeeding period.

Supplementary Table S12 shows the logistic regression analysis of the associations between prenatal PCB, PFAS and metal exposure and the risk of abnormal hyperactivity score. In the analyses with continuous exposure variables, PCB118 (Model 1 OR = 1.10, *p* = 0.085, Model 2 OR: 1.10, *p* = 0.088), and ∑PCB (Model 1 OR = 1.01, *p* = 0.096), were borderline significant associated with an increased risk of abnormal hyperactivity score. PFOA was inversely associated with abnormal hyperactivity score in all models (unadjusted OR = 0.09, *p* = 0.078; Model 1 OR = 0.08, *p* = 0.073; Model 2 OR = 0.09, *p* = 0.094). Finally, none of the metal exposure variables had any significant association to abnormal hyperactivity score (Supplementary Table S12).

In the analyses with continuous exposure variables stratified by gender, the statistical power was markedly reduced due to the low number of children with abnormal hyperactivity scores (boys n = 4, girls n = 1) (Supplementary Table S13). Most results were similar among boys and girls and non-significant, although the association with PFOA was inverse and borderline significantly among boys (Model 1 OR = 0.06, *p* = 0.082), whereas the association with Se was weakly positive and borderline significant among girls (unadjusted OR = 1.08, *p* = 0.074) (Supplementary Table S13).

### Sensitivity analyses

In addition to adjustment model 2 and gender stratification, being presented in the text and tables, sensitivity analyses including BMI, marine intake (n-3/n-6) and GA as confounders in the models were performed (data not shown). The results from the sensitivity analyses were generally similar with the presented results and did not affect the conclusion (data not shown).

## Discussion

This study presents follow up data of 102 Greenlandic Inuit mother–child pairs from Nuuk, Sisimiut and Ilulissat, included in the ACCEPT cohort. Children were followed up at the age of 3–5 years and behavior problems were assessed with the SDQ. The percentage of children with abnormal SDQ score (12%) were higher than previously reported among older children (5–9 years) in Greenland (6%), whereas the children with abnormal hyperactivity score were similar with previous reported data (5% in present study versus 4% previously)^52,53^. We found that prenatal POP exposure, especially OCPs, were associated with problematic child behavior (high/abnormal SDQ and hyperactivity scores). Although the direction for a few associations were not consistent; high maternal POP and metals levels during pregnancy were associated with lower SDQ and hyperactivity scores. The gender stratified data, even though with low statistical power, indicate that boys are more susceptible than girls.

In this study, a significant higher concentrations of almost all POPs and metals were observed in Sisimiut/Ilulissat compared to Nuuk. This might be explained by differences in lifestyle, with higher intake of traditional diet consisting of more predatory fish and marine mammals in Sisimiut/Ilulissat, in addition to a higher smoking prevalence in Sisimiut/Ilulissat (30.3%) compared to Nuuk (15.9%), since smoking can contribute to the body level of POPs and metals^74^. Differences in food intake between regions in Greenland have been observed before, both in the ACCEPT cohort as well as in other studies^27,28,75,76^. Differences in smoking have also been observed^27,28^. ΣOCP, ΣLegacyPOPs, Pb and Cd were significantly positively correlated with plasma cotinine levels (indication of current smoking) in the present study, which have also been seen previously in the ACCEPT cohort^35,36^. All the sums of lipophilic POPs were positively correlated with age and negatively correlated with BMI, which can be explained by their long half-lives in the body^77^ and accumulation in adipose tissue^78^, respectively. Sea food is the primary source of human exposure to POP, which were supported by the positive correlations between n-3/n-6 ratio (biomarker for sea food intake) and POP concentrations seen both in the present study and previously in the ACCEPT cohort^35^. Cd was negatively correlated to n-3/n-6 ratio suggesting an inverse relation between marine food intake and Cd level (Supplementary Table S3).

Most of the investigated OCPs in the present study showed significant positive associations and OR’s with high/abnormal SDQ score, whereas, Mirex was inversely significant associated with hyperactivity scores in the linear regression analysis. A Flemish cohort study including 1196 Flemish mother–child pairs did not find any association between prenatal Hexachlorobenzene exposure and abnormal SDQ score at 7–8 years of age, however a significant positive association between prenatal p,p’-DDE exposure and abnormal SDQ score was found in girls^57^. The different associations observed between the Greenlandic and Flemish population might relate to the difference in biological samples (early pregnancy blood vs. cord blood), Hexachlorobenzene level (27.0 µg/kg lipid vs 22.6 µg /kg lipid) and child age (3–5 years vs 7–8 years). In addition, a study, including 1018 mother–child pairs from Greenland and Ukraine aged 5–9 years showed a significant positive association between p,p’-DDE and abnormal SDQ score^58^. A number of prospective studies have also reported that prenatal exposure to OCPs may be associated with other neurodevelopmental and neurobehavioral defects in the off-spring^79^.

Generally, the maternal PCBs concentration were not significantly associated with either SDQ or hyperactivity score in the present study. Although, positive and significant ORs were seen for PCB118 and ∑PCBs and abnormal hyperactivity scores. Few previous studies have assessed prenatal PCB exposure and problematic behavior measured by SDQ. A Greenlandic and Ukrainian study, found prenatal PCB153 exposure to be associated with the risk of child abnormal hyperactivity scores, whereas they did not find an association with abnormal SDQ scores at the age of 5–9 years of age^58^. The Flemish study showed no association between PCBs (PCB118, PCB138, PCB153, PCB170, PCB180) and SDQ score or sub-scores at 7–8 years of age^57^.

The present study did not find any positive associations between PFASs and SDQ or hyperactivity score, however PFOA and ∑PFCA were inversely associated with hyperactivity scores/abnormal hyperactivity scores. In contrast, another study found a significant positive association between prenatal PFOS exposure and SDQ score and prenatal PFOA and hyperactivity score at age 5–9 years among 1106 mother–child pairs from Ukraine, Poland and Greenland^52^. In the same cohort study, among the Greenlandic and Ukrainian mother–child pairs (n = 1023), a significant positive association between prenatal PFHxS and PFNA exposure and SDQ score were seen, whereas no consistent associations for prenatal PFHxA or PFDA exposure were found^53^.

In the present study prenatal Hg and Se exposure were significantly inversely associated with continuous SDQ score. While the direction of the Hg association was unexpected, it was hypnotized that the trace element Se, which is correlated to marine intake, was inversely associated with SDQ scores. Maternal intake of non-predatory fish and seafood (except marine mammals) during pregnancy have in several studies been associated with beneficial neurocognitive development in children^80^, for instance children of mothers eating oily seafood in early pregnancy had lower risk of hyperactivity and abnormal SDQ scores at the age of 9 years in England^81^. The inverse association found for Hg may be a result of residual confounding as marine intake is the primary source of Hg exposure. To account for the strong relationship between marine intake and POP/metal exposure, we did include n-3/n-6 ratio, as a biomarker of marine food, as a confounder in additional sensitivity analyses (data not shown). The results were of similar strength and significance, thus the conclusions remained the same.

A major source of postnatal POP exposure is breastfeeding^82–84^. Thus, in order to isolate the effect of prenatal exposure we adjusted for breastfeeding duration in adjustment model 2. The results were generally similar, however, adjustment for breastfeeding duration did in some cases seem to attenuate the associations slightly for the SDQ scores. This indicate that the associations in part are explained by postnatal exposure through breastfeeding or by other beneficial effects of breastfeeding. Breastfeeding have been associated with beneficial child behavior independently in some^85,86^, but not all^84^, studies.

When stratifying for gender, the association between prenatal POP and metal exposure and SDQ and hyperactivity score were generally similar among boys and girls. For the associations between prenatal OCP exposure and SDQ score, the associations were somewhat stronger and more significant among the boys. A possible explanation could be that boys may be more sensitive to prenatal OCP exposure than girls. However, this is in contrast to the Flemish study, which found an association between prenatal p,p’-DDE exposure and abnormal SDQ score in girls, and not boys^57^. However, it should be noted the statistical significance in the present study dropped markedly because of the low number of participants (boys n = 55/girls n = 47), thus the results may only been seen as hypothesis-generating for future larger studies.

The children in the present study are younger (3–5 years) than in previous studies on prenatal POP exposure and SDQ score, 5–9 years^52,53,58^ and 7–8 years^57^. The SDQ is appropriate for children aged 3–16 years, but one could speculate that it is harder for the parents to identify problematic behavior in an early age and that some children not yet have developed problematic behavior. This might explain some of the differences in results seen, together with differences in exposure levels.

Several strengths and limitations of the study should be taken into account when evaluating the results*.* Compared to other similar studies, the study population in the present study was small (N = 102), limiting the statistical power to detect weak associations between prenatal POP and metal exposure and SDQ or hyperactivity score. Especially in the logistic regression analyses, which only include 11 cases with abnormal SDQ score and 5 cases with abnormal hyperactivity score. We did try to group the borderline and abnormal cases together to increase the number; however, it may weaken the results due to misclassification. We did not see any significant associations with SDQ or hyperactivity when borderline and abnormal were grouped together (data not shown). To be able to compare our results with previous studies^52,53^, we did use the same grouping with normal/borderline versus abnormal despite the small number of cases.

The participation rate was high (76.6%) and the missing data in the outcome variables was relatively low (SDQ score: 6.9%; hyperactivity score: 1.0%). Compared to other studies on prenatal POP exposure and SDQ scores, the number of exposure compounds in the present study were large. Although it is known that POPs are highly inter-correlated and there can be some interaction between POPs^87–89^, it is a great advantage that several chemicals groups have been analyzed in the same study population, to compare the effects of the different compounds. However, due to the many compounds and, thus, analyses, it is important to note that some of significant associations found in the present study could be due to chance. To address this issue, we adjusted for the false discovery rate (FDR) using the Benjamini–Hochberg procedure^90,91^. The FDR correction did not change the significance levels (data not shown). In addition, the other potential covariates such as weight gain of pregnant women, father’s characteristics and serious infection of child^57^ were not considered in the present study due to these data were unavailable.

Child behavior was assessed using the SDQ, which is a valid screening tool on population level^73,92,93^. It should be noted that even small changes in SDQ score on the individual level, could be problematic when looking at a whole population. It is, however, important to note that the SDQ is not a diagnostic tool and that psychiatrists may have been able to provide a more valid evaluation, but that would not have been cost-effective. Similar questionnaires with SDQ have been used in Greenland before^52,53^, and were provided in Danish and Greenlandic and filled out by the parents in cooperation with a health professional. This may have eliminated possible uncertainties about the meaning of the questions, however, it cannot be ruled out that some of the parents did not understand all questions perfectly well. The fact that the parents were not anonymous had the potential to bias the answers. Parents of children with problematic behavior could have underreported the problems (intentionally or unintentionally), whereas parents of children without problematic behavior would not have any reason to do so. This could have drawn the associations towards the null. However, as more children had an abnormal SDQ score in the present study (12%) than previously reported in Greenland (6%)^52,53^, indicates that this might not be a problem.

## Conclusion

In the present study, we found a higher percentages of children (3–5 years) with abnormal SDQ score (12%) than previously reported in older children in Greenland (6%)^52,53^. We investigated the associations between prenatal exposure to OCP, PCB, PFAS and metal and SDQ and hyperactivity scores. For most OCPs, we found significant positive associations with SDQ and hyperactivity score. While for the PCBs and PFASs, less consistent negative and positive associations were seen. Results from gender stratified analyses suggested that boys may be more sensitive to the exposure than girls. This study indicate that prenatal POP exposure (especially OCPs) could be linked to problematic behavior in children aged 3–5 years, however, studies on larger populations are needed in order to strengthen the evidence.

## Supplementary Information

Supplementary Information.
