# Publisher Correction: Prenatal exposure to persistent organic pollutants and metals and problematic child behavior at 3–5 years of age: a Greenlandic cohort study

**DOI:** 10.1038/s41598-021-03923-3

**Published:** 2021-12-21

**Authors:** Simon Kornvig, Maria Wielsøe, Manhai Long, Eva Cecilie Bonefeld-Jørgensen

**Affiliations:** 1 Center for Arctic Health and Molecular Epidemiology, Department of Public Health, Aarhus University, Aarhus, Denmark; 2 Greenland Centre for Health Research, University of Greenland, Nuuk, Greenland

Correction to: *Scientific Reports* 10.1038/s41598-021-01580-0, published online 12 November 2021

The original version of this Article contained errors.

Table 4 and Table 5 contained several errors in the data given for rows “Cont”, “Low”, “High” and “p-trend” due to an incorrect merging of cells.

Additionally, in Table 5, under the subheading for compound “cis-Nonachlor”, p-values for the row “Cont” and ß (95% CI) values for the row “High” were omitted.

The original Table 4 and 5 and accompanying legends appear below.

**Table 4:** Linear regression analysis of associations between prenatal OCP exposure and continuous SDQ score: Greenlandic children 3–5 years of age born 2014–2016 in the ACCEPT birth cohort.

All (n = 95)						
OCPs (µg/kg lipid)	Unadjusted		Adjustment model 1		Adjustment model 2	
	β (95% CI)	*p*-Value	β (95% CI)	*p*-Value	β (95% CI)	*p*-Value
**cis-Nonachlor**						
Cont	0.05 (− 0.11, 0.21)	0.533	0.07 (− 0.10, 0.24)	0.403	0.06 (− 0.11, 0.24)	0.457
Low	Ref		Ref		Ref	
Med	0.05 (− 2.45, 2.56)	0.967	1.38 (− 1.01, 3.77)	0.258	1.17 (− 1.29, 3.63)	0.351
High	0.18 (− 2.75, 2.38)	0.889	1.36 (− 1.13, 3.84)	0.286	1.22 (− 1.32, 3.76)	0.346
p-trend		0.892		0.795		0.938
**Hexachlorobenzene**						
Cont	0.02 (− 0.04, 0.07)	0.543	0.02 (− 0.04, 0.07)	0.566	0.01 (− 0.04, 0.07)	0.641
Low	Ref		Ref		Ref	
Med	1.91 (− 0.59, 4.40)	0.134	3.06 (0.74, 5.39)	0.010*	2.95 (0.60, 5.30)	0.014*
High	0.31 (− 2.21, 2.82)	0.812	1.71 (− 0.68, 4.10)	0.161	1.59 (− 0.83, 4.01)	0.197
p-trend		0.805		0.555		0.668
**Mirex**						
Cont	− 0.11 (− 0.51, 0.29)	0.572	− 0.12 (− 0.53, 0.29)	0.557	− 0.14 (− 0.55, 0.27)	0.503
Low	Ref		Ref		Ref	
Med	− 1.88 (− 4.34, 0.58)	0.134	− 0.66 (− 3.16, 1.85)	0.608	− 0.97 (− 3.55, 1.61)	0.462
High	− 1.14 (− 3.64, 1.37)	0.374	0.17 (− 2.31, 2.65)	0.892	− 0.01 (− 2.49, 2.46)	0.991
p-trend		0.351		0.444		0.385
**Oxychlordane**						
Cont	0.03 (− 0.03, 0.09)	0.404	0.03 (− 0.04, 0.09)	0.415	0.02 (− 0.04, 0.08)	0.475
Low	Ref		Ref		Ref	
Med	− 0.52 (− 3.00, 1.97)	0.684	0.78 (− 1.64, 3.20)	0.528	0.56 (− 1.89, 3.02)	0.654
High	0.28 (− 2.28, 2.85)	0.829	1.57 (− 0.88, 4.01)	0.209	1.42 (− 1.07, 3.91)	0.262
p-trend		0.849		0.630		0.757
**p,p’-DDE**						
Cont	0.01 (− 0.01, 0.02)	0.330	0.01 (− 0.01, 0.02)	0.322	0.01 (− 0.01, 0.02)	0.368
Low	Ref		Ref		Ref	
Med	− 0.06 (− 2.55, 2.43)	0.962	0.42 (− 2.03, 2.86)	0.738	0.22 (− 2.29, 2.72)	0.866
High	0.66 (− 1.95, 3.26)	0.621	1.93 (− 0.58, 4.45)	0.132	1.74 (− 0.81, 4.28)	0.180
p-trend		0.631		0.422		0.519
**ß-HCH**						
Cont	0.18 (− 0.22, 0.58)	0.371	0.26 (− 0.17, 0.68)	0.231	0.24 (− 0.19, 0.67)	0.264
Low	Ref		Ref		Ref	
Med	2.20 (− 0.29, 4.68)	0.083**	3.58 (1.16, 6.00)	0.004*	3.51 (1.04, 5.98)	0.005*
High	0.48 (− 2.04, 3.01)	0.707	2.03 (− 0.39, 4.45)	0.100**	1.95 (− 0.53, 4.43)	0.123
p-trend		0.715		0.419		0.523
**trans-Nonachlor**						
Cont	0.01 (− 0.02, 0.04)	0.418	0.02 (− 0.01, 0.04)	0.319	0.01 (− 0.02, 0.04)	0.366
Low	Ref		Ref		Ref	
Med	0.68 (− 1.79, 3.14)	0.590	2.06 (− 0.26, 4.39)	0.082**	1.90 (− 0.48, 4.27)	0.117
High	− 0.11 (− 2.70, 2.48)	0.935	1.61 (− 0.91, 4.14)	0.210	1.52 (− 1.01, 4.09)	0.249
p-trend		0.962		0.660		0.788
**∑OCPs**						
Cont	0.00 (− 0.00, 0.01)	0.377	0.00 (− 0.00, 0.01)	0.349	0.00 (− 0.00, 0.01)	0.400
Low	Ref		Ref		Ref	
Med	0.97 (− 1.51, 3.45)	0.443	1.97 (− 0.46, 4.41)	0.113	1.83 (− 0.65, 4.31)	0.149
High	0.18 (− 2.38, 2.74)	0.889	1.36 (− 1.06, 3.78)	0.270	1.22 (− 1.26, 3.69)	0.335
p-trend		0.868		0.650		0.780

*n* Number of participants in parameter, *β* Linear regression coefficient in score points, *CI* confidence interval, Bold text and * = Significant (*p* ≤ 0.050), Bold text and ** = Borderline significant (*p* ≤ 0.100), Adjustment model 1: Maternal plasma cotinine, maternal educational level, maternal age at delivery, Adjustment model 2: Adjustment model 1 + breast-feeding duration, Med = Medium, Cont. = Continuous, *OCPs* organochlorine pesticides.

**Table 5:** Linear regression analysis of the associations between prenatal OCP exposure and continuous hyperactivity score: Greenlandic children 3–5 years of age born 2014–2016, the ACCEPT birth cohort.

All (n = 101)						
OCPs (µg/kg lipid)	Unadjusted		Adjustment model 1		Adjustment model 2	
	β (95% CI)	*p*-Value	β (95% CI)	*p*-Value	β (95% CI)	*p*-Value
**cis-Nonachlor**						
Cont	0.03 (− 0.03, 0.09)	0.279	0.03 (− 0.03, 0.09)		0.03 (− 0.03, 0.10)	
Low	Ref		Ref		Ref	
Med	− 0.49 (− 1.38, 0.41)	0.287	− 0.44 (− 1.33, 0.46)	0.341	− 0.40 (− 1.33, 0.53)	0.398
High		0.975		0.686		0.651
p-trend		0.967		0.967		0.884
**Hexachlorobenzene**						
Cont	0.00 (− 0.02, 0.02)	0.797	0.00 (− 0.02, 0.02)	0.873	0.00 (− 0.02, 0.02)	0.814
Low	Ref		Ref		Ref	
Med	− 0.20 (− 1.09, 0.70)	0.665	− 0.24 (− 1.13, 0.65)	0.603	− 0.22 (− 1.13, 0.68)	0.627
High	− 0.09 (− 1.00, 0.82)	0.844	0.08 (− 0.85, 1.00)	0.874	0.09 (− 0.85, 1.04)	0.848
p-trend		0.846		0.822		0.887
**Mirex**						
Cont	− 0.01 (− 0.15, 0.13)	0.882	− 0.02 (− 0.17, 0.13)	0.807	− 0.02 (− 0.16, 0.13)	0.843
Low	Ref		Ref		Ref	
Med	− 1.01 (− 1.87, − 0.14)	0.023*	− 1.18 (− 2.08, − 0.28)	0.010*	− 1.35 (− 2.23, − 0.43)	0.004*
High	− 0.26 (− 1.12, 0.61)	0.561	− 0.14 (− 1.02, 0.75)	0.766	− 0.23 (− 1.12, 0.66)	0.616
p-trend		0.517		0.447		0.467
**Oxychlordane**						
Cont	0.01 (− 0.01, 0.03)	0.234	0.01 (− 0.01, 0.04)	0.271	0.01 (− 0.01, 0.04)	0.246
Low	Ref		Ref		Ref	
Med	− 0.56 (− 1.44, 0.32)	0.215	− 0.71, (− 1.60, 0.19)	0.121	− 0.71 (− 1.62, 0.21)	0.129
High	0.14 (− 0.75, 1.03)	0.764	0.23 (− 0.66, 1.11)	0.617	0.25 (− 0.65, 1.16)	0.585
p-trend		0.910		0.973		0.946
**p,p’-DDE**						
Cont	0.00 (− 0.00, 0.01)	0.237	0.00 (− 0.00. 0.01)	0.284	0.00 (− 0.00, 0.01)	0.256
Low	Ref		Ref		Ref	
Med	− 0.32 (− 1.20, 0.56)	0.476	− 0.59 (− 1.50, 0.32)	0.205	− 0.59 (− 1.53, 0.35)	0.217
High	0.06 (− 0.86, 0.98)	0.895	0.10 (− 0.83, 1.04)	0.830	0.13 (− 0.82, 1.08)	0.788
p-trend		0.780		0.853		0.776
**ß-HCH**						
Cont	0.08 (− 0.06, 0.22)	0.269	0.08 (− 0.07, 0.23)	0.294	0.09 (− 0.07, 0.25)	0.260
Low	Ref		Ref		Ref	
Med	0.50 (− 0.39, 1.39)	0.270	0.55 (− 0.37, 1.48)	0.240	0.65 (− 0.29, 1.82)	0.177
High	0.22 (− 0.68, 1.11)	0.637	0.46 (− 0.48, 1.39)	0.337	0.53 (− 0.43, 1.49)	0.278
p-trend		0.637		0.650		0.566
**trans-Nonachlor**						
Cont	0.01 (− 0.00, 0.02)	0.208	0.01 (− 0.00, 0.02)	0.231	0.01 (− 0.00, 0.02)	0.209
Low	Ref		Ref		Ref	
Med	− 0.50 (− 1.38, 0.38)	0.265	− 0.48 (− 1.35, 0.40)	0.285	− 0.44 (− 1.34, 0.45)	0.334
High	0.19 (− 0.71, 1.08)	0.685	0.35 (− 0.58, 1.28)	0.458	0.39 (− 0.55, 1.34)	0.416
p-trend		0.712		0.725		0.649
**∑OCPs**						
Cont	0.00 (− 0.00, 0.00)	0.265	0.00 (− 0.00, 0.00)	0.309	0.00 (− 0.00, 0.00)	0.278
Low	Ref		Ref		Ref	
Med	− 0.32 (− 1.21, 0.56)	0.473	− 0.56 (− 1.47, 0.34)	0.223	− 0.52 (− 1.45, 0.41)	0.271
High	0.34 (− 0.55, 1.24)	0.449	0.37 (− 0.52, 1.26)	0.415	0.40 (− 0.51, 1.31)	0.391
p-trend		0.466		0.526		0.457

*n* Number of participants in parameter, *β* Linear regression coefficient in score points, *CI* confidence interval, Bold text and *= Significant (*p* ≤ 0.050), Bold text and ** = Borderline significant (*p* ≤ 0.100), Adjustment model 1: Maternal plasma cotinine, maternal educational level, maternal age at delivery, Adjustment model 2: Adjustment model 1 + breast-feeding duration, Med = Medium, Cont. = Continuous, *OCPs* organochlorine pesticides.

The original Article has been corrected.

