# Supplementary material for: Prenatal exposure to persistent organic pollutants and metals and problematic child behavior at 3–5 years of age: a Greenlandic cohort study
Source: Sci Rep. 2021 Nov 12;11:22182. doi: 10.1038/s41598-021-01580-0 (PMC8589846; doi:10.1038/s41598-021-01580-0)
Supplement: Supplementary file 1 — Supplementary Information. [file 41598_2021_1580_MOESM1_ESM.pdf]

SUPPLEMENTARY TABLES

Prenatal exposure to Persistent Organic Pollutants and Metals and Problematic Child Behavior at 3-5 Years of Age: A Greenlandic cohort study

Simon Kornvig[1], Maria Wielsøe [1], Manhai Long[1], and Eva Cecilie Bonefeld-Jørgensen [1,2]

[1] Center for Arctic Health and Molecular Epidemiology, Department of Public Health, Aarhus University, Denmark, [2] Greenland Centre for Health Research, University of Greenland, Greenland

Table S1: Included POPs and sums of POPs

∑PCBs	∑OCPs	∑LegacyPOPs	∑PBDEs	∑LipophilicPOPs	∑PFSAs	∑PFCAs	∑PFASs
PCB 101	Aldrin	∑PCB	PBDE 100	∑PCB	PFBS	PFPeA	∑PFSA
PCB 105	alpha-Chlordane	∑OCP	PBDE 15	∑OCP	PFHxS	PFHxA	∑PFCA
PCB 118	cis-Nonachlor		PBDE 153	∑PBDE	PFHpS	PFHpA	
PCB 128	gamma-Chlordane		PBDE 17		PFOS	PFOA	
PCB 138	Hexachlorobenzene		PBDE 25		PFDS	PFNA	
PCB 153	Mirex		PBDE 28		PFOSA	PFDA	
PCB 156	Oxychlordane		PBDE 33			PFUnA	
PCB 170	p,p'-DDE		PBDE 47			PFDoA	
PCB 180	p,p'-DDT		PBDE 99			PFTrA	
PCB 183	ß-HCH		PBB 153			PFTeA	
PCB 187	trans-Nonachlor						
PCB 28							
PCB 52							
PCB 99							

PCB: polychlorinated biphenyl, OCP: organochlorine pesticides, p,p'-DDE: dichlorodiphenyltrichloroethane, p,p'-DDT: dichlorodiphenyldichloroethylene, ß-HCH: β-hexachlorocyclohexane, PBDE: polybrominated diphenyl ether, PBB: polybrominated biphenyl, PFSA: Perfluoroalkylated substances, PFBS: perfluorobutanesulfonic acid, PFHxS: perfluorohexane sulfonate, PFHpS: perfluoroheptanesulfonate, PFOS: perfluorooctane sulfonate, PFDS: perfuoro-1-decanesulfonate, PFOSA: perfluorooctane sulfonamide, PFCA: perfluorinated carboxylic acid, PFPeA: perfluoropentanoic acid, PFHxA: Perfluorohexanoic acid, PFHpA: perfluoroheptanoic acid, PFOA: perfluorooctanoic acid, PFNA: perfluorononanoic acid, PFDA: perfluorodecanoic acid, PFUnA: perfluoroundecanoic acid, PFDoA: perfluorododecanoic acid, PFTrA: perfluorotridecanoic acid, PFTeA: perfluorotetradecanoic acid. PFAS: Perfluoroalkylated Substances

Table S2: Spearman correlation coefficient (r_s) between the sums of POPs and metals: Greenlandic pregnant women in the ACCEPT cohort 2013-2015.

	∑PCBs ⅄	∑OCPs ⅄	∑Legacy POPs ⅄	∑LipPOPs ⅄	∑PFSAs ✦	∑PFCAs ✦	∑PFASs ✦	Hg★	Pb★	Cd★	Se★
∑PCBs ⅄											
∑OCPs ⅄	**0.921*** **										
∑LegacyPOPs ⅄	**0.968*** **	**0.986*** **									
∑LipPOPs ⅄	**0.967*** **	**0.985*** **	**0.998*** **								
∑PFSAs ✦	**0.654*** **	**0.725*** **	**0.709*** **	**0.717*** **							
∑PFCAs ✦	**0.391*** **	**0.507*** **	**0.464*** **	**0.470*** **	**0.693*** **						
∑PFASs ✦	**0.549*** **	**0.652*** **	**0.620*** **	**0.624*** **	**0.917*** **	**0.908*** **					
Hg★	**0.401*** **	**0.452*** **	**0.429*** **	**0.428*** **	**0.475*** **	**0.403*** **	**0.453*** **				
Pb★	**0.305*** **	**0.272*** **	**0.282*** **	**0.266*** **	**0.235** **	**0.212** **	**0.230** **	0.084			
Cd★	**0.230** **	**0.209** **	**0.227** **	**0.187* **	**0.197** **	0.098	0.139	**0.177* **	**0.282*** **		
Se★	**0.436*** **	**0.498*** **	**0.473*** **	**0.487*** **	**0.424*** **	**0.363*** **	**0.415*** **	**0.698*** **	**0.222** **	0.084	

⅄: (µg/kg lipid), ✦: (ng/mL), ★: (µg/L), ***: Highly significant (p ≤0.010), **: Significant (p ≤0.050), *: Borderline significant (p ≤0.100), ∑LipPOPs = ∑LipophilicPOPs, PCB: polychlorinated biphenyl, OCPs: Organochlorine pesticides, PFSA: Perfluoroalkylated substances, PFCA: perfluorinated carboxylic acid, PFAS: Perfluoroalkylated

Table S3: Spearman correlation coefficient (r_s) between the sums of POPs, metals and lifestyle factors: Greenlandic pregnant women in the ACCEPT cohort 2013-2015.

	Age (years)			BMI (kg/m²)			P-cotinine (ng/mL)			n-3/n-6		
	n	r_s	p	n	r_s	p	n	r_s	p	n	r_s	p
∑PCBs ⅄	102	0.262	**0.008***	100	-0.223	**0.044***	102	0.119	0.233	101	0.126	0.210
∑OCPs ⅄	102	0.215	**0.030***	100	-0.193	**0.054****	102	0.197	**0.048***	101	0.183	**0.067****
∑LegacyPOPs ⅄	102	0.235	**0.017***	100	-0.203	**0.043***	102	0.172	**0.084****	101	0.159	0.113
∑LipophilicPOPs ⅄	97	0.213	**0.036***	95	-0.238	**0.020***	97	0.150	0.142	96	0.169	**0.099****
∑PFSAs ✦	102	0.063	0.532	100	-0.025	0.806	102	0.092	0.358	101	0.252	**0.011***
∑PFCA's ✦	102	-0.068	0.497	100	-0.046	0.648	102	0.100	0.316	101	0.259	**0.009***
∑PFAS's ✦	102	-0.015	0.852	100	-0.019	0.852	102	0.089	0.374	101	0.293	**0.003***
Hg ★	102	0.158	0.114	100	0.060	0.552	102	0.096	0.337	101	0.084	0.402
Pb ★	102	0.079	0.431	100	0.027	0.792	102	0.292	**0.003***	101	0.121	0.228
Cd ★	102	0.083	0.408	100	0.023	0.821	102	0.544	**<0.001***	101	-0.182	**0.068****
Se ★	102	0.118	0.237	100	-0.073	0.473	102	0.071	0.476	101	0.255	**0.010***

n: number of participants for actual analyses; ⅄: (µg/kg lipid), ✦: (ng/mL), ★: (µg/L), *: Significant (p ≤0.050), **: Borderline significant (p ≤ 0.100), PCB: polychlorinated biphenyl, OCPs: Organochlorine pesticides, , PFSA: Perfluoroalkylated substances, PFCA: perfluorinated carboxylic acid, PFAS: Perfluoroalkylated Substances

Table S4: Linear regression analysis of associations between prenatal POP and metal exposure and continuous SDQ score: Greenlandic children 3-5 years of age born 2014-2016 in the ACCEPT birth cohort.

All (n = 95)			Unadjusted		Adjustment model 1		Adjustment model 2	
			β (95% CI)	p-Value	β (95% CI)	p-Value	β (95% CI)	p-Value
**PCBs**	PCB118	Cont.	0.04 (-0.11, 0.18)	0.604	0.06 (-0.10, 0.21)	0.480	0.05 (-0.11, 0.20)	0.544
		Low	Ref.		Ref.		Ref.	
**(μg/kg lipid)**		Med	0.88 (-1.67, 3.43)	0.500	1.68 (-0.77, 4.14)	0.179	1.47 (-1.04, 3.98)	0.252
		High	-0.64 (-3.13, 1.85)	0.613	1.04 (-1.50, 3.58)	0.421	0.83 (-1.81, 3.46)	0.538
		p-trend		0.614		0.956		0.811
	PCB138	Cont.	0.01 (-0.04, 0.06)	0.717	0.01 (-0.05, 0.07)	0.697	0.01 (-0.05, 0.06)	0.773
		Low	Ref.		Ref.		Ref.	
		Med	0.90 (-1.59, 3.40)	0.478	1.92 (-0.47, 4.31)	0.116	1.70 (-0.73, 4.14)	0.171
		High	-0.01 (-2.55, 2.53)	0.994	1.60 (-0.89, 4.08)	0.209	1.44 (-1.11, 3.98)	0.298
		p-trend		0.988		0.643		0.755
	PCB153	Cont.	0.00 (-0.02, 0.02)	0.960	0.00 (-0.02, 0.02)	0.977	-0.00 (-0.03, 0.02)	0.942
		Low	Ref.		Ref.		Ref.	
		Med	-1.07 (-3.53, 1.39)	0.393	-0.07 (-2.51, 2.38)	0.957	-0.29 (-2.76, 2.17)	0.812
		High	-0.81 (-3.41, 1.79)	0.543	0.83 (-1.72, 3.38)	0.526	0.58 (-1.99, 3.15)	0.658
		p-trend		0.533		0.876		0.773
	PCB156	Cont.	-0.15 (-0.52, 0.21)	0.405	-0.10 (-0.47, 0.27)	0.584	-0.12 (-0.49, 0.25)	0.521
		Low	Ref.		Ref.		Ref.	
		Med	-1.03 (-3.54, 1.47)	0.419	0.18 (-2.39, 2.74)	0.892	-0.10 (-2.67, 2.47)	0.939
		High	-1.52 (-4.06, 1.03)	0.243	0.28 (-2.29, 2.85)	0.831	0.05 (-2.54, 2.62)	0.973
		p-trend		0.215		0.546		0.450
	PCB170	Cont.	-0.05 (-0.19, 0.09)	0.505	-0.03 (-0.18, 0.11)	0.639	-0.04 (-0.18, 0.11)	0.589
		Low	Ref.		Ref.		Ref.	
		Med	-2.88 (-5.29, -0.46)	**0.019***	-1.72 (-4.24, 0.81)	0.182	-1.93 (-4.45, 0.60)	0.135
		High	-2.18 (-4.70, 0.33)	**0.089****	-0.68 (-3.25, 1.89)	0.605	-0.86 (-3.42, 1.69)	0.508
		p-trend		**0.091****		0.225		0.184
	PCB180	Cont.	-0.01 (-0.06, 0.03)	0.592	-0.01 (-0.06, 0.04)	0.685	-0.01 (-0.06, 0.04)	0.631
		Low	Ref.		Ref.		Ref.	
		Med	-1.46 (-3.95, 1.03)	0.249	-0.29 (-2.83, 2.25)	0.822	-0.54 (-3.07, 2.00)	0.678
		High	-1.95 (-4.54, 0.65)	0.142	-0.13 (-2.77, 2.52)	0.926	-0.32 (-2.97, 2.32)	0.811
		p-trend		0.151		0.366		0.296
	PCB183	Cont.	-0.07 (-0.52, 0.39)	0.772	-0.04 (-0.52, 0.45)	0.885	-0.06 (-0.55, 0.43)	0.806
		Low	Ref.		Ref.		Ref.	
		Med	-1.12 (-3.59, 1.34)	0.372	0.28 (-2.24, 2.81)	0.826	0.09 (-2.44, 2.62)	0.946
		High	-0.80 (-3.42, 1.82)	0.550	0.77 (-1.91, 3.45)	0.547	0.63 (-2.04, 3.30)	0.642
		p-trend		0.543		0.960		0.876
	PCB187	Cont.	-0.01 (-0.12, 0.09)	0.800	-0.01 (-0.12, 0.11)	0.919	-0.01 (-0.12, 0.10)	0.850
		Low	Ref.		Ref.		Ref.	
		Med	0.42 (-2.08, 2.93)	0.741	1.68 (-0.71, 4.07)	0.169	1.45 (-0.97, 3.87)	0.240
		High	0.00 (-2.56, 2.57)	0.997	1.40 (-1.07, 3.86)	0.268	1.27 (-1.21, 3.75)	0.314
		p-trend		0.992		0.686		0.788
	PCB99	Cont.	0.07 (-0.08, 0.21)	0.382	0.07 (0.09, 0.23)	0.372	0.06 (-0.10, 0.22)	0.431
		Low	Ref.		Ref.		Ref.	
		Med	0.92 (-1.64, 3.48)	0.482	0.84 (-1.63, 3.31)	0.504	0.67 (-1.84, 3.17)	0.601
		High	0.06 (-2.42, 2.54)	0.962	0.74 (-1.72, 3.19)	0.555	0.57 (-1.91, 3.05)	0.653
		p-trend		0.962		0.814		0.937
	**∑PCBs**	Cont.	-0.00 (-0.01, 0.01)	0.873	0.00 (-0.01, 0.01)	0.930	-0.00 (-0.01, 0.01)	0.845
		Low	Ref.		Ref.		Ref.	
		Med	-2.23 (-4.68, 0.22)	**0.074****	-1.36 (-3.74, 1.22)	0.321	-1.47 (-3.96, 1.02)	0.246
		High	-1.30 (-3.85, 1.24)	0.316	0.23 (-2.38, 2.84)	0.862	0.05 (-2.56, 2.66)	0.971
		p-trend		0.309		0.600		0.515
	**∑LegacyPOPs**	Cont.	0.00 (-0.00, 0.00)	0.643	0.00 (-0.00, 0.01)	0.596	0.00 (-0.00, 0.01)	0.670
		Low	Ref.		Ref.		Ref.	
		Med	0.80 (-1.68, 3.28)	0.528	1.56 (-0.82, 3.95)	0.199	1.38 (-1.07, 3.83)	0.269
		High	0.06 (-2.52, 2.64)	0.964	1.55 (-0.95, 4.05)	0.223	1.43 (-1.11, 3.96)	0.269
		p-trend		0.949		0.664		0.789

	**∑LipophilicPOPs**	Cont.	0.00 (-0.00, 0.00)	0.671	0.00 (-0.00, 0.01)	0.561	0.00 (-0.00, 0.01)	0.609
		Low	Ref.		Ref.		Ref.	
		Med	0.65 (-1.90, 3.20)	0.618	1.57 (-0.93, 4.07)	0.219	1.39 (-1.18, 3.96)	0.228
		High	-0.19 (-2.85, 2.47)	0.890	1.40 (-1.16, 3.95)	0.284	1.27 (-1.32, 3.85)	0.337
		p-trend		0.902		0.739		0.829
**PFSAs (ng/mL)**	PFHxS	Cont.	-3.18 (-8.18, 1.83)	0.210	-2.97 (-8.14, 2.20)	0.256	-3.58 (-9.02, 1.86)	0.195
		Low	Ref.		Ref.		Ref.	
		Med	0.85 (-1.64, 3.35)	0.502	1.23 (-1.13, 3.58)	0.307	1.59 (-0.81, 3.98)	0.194
		High	-1.59 (-4.07, 0.88)	0.207	-0.97 (-3.36, 1.42)	0.425	-0.97 (-3.45, 1.51)	0.442
		p-trend		0.220		0.266		0.237
	PFHpS	Cont.	-6.33 (-18.2, 5.50)	0.291	-6.58 (-18.5, 5.34)	0.276	-9.08 (-21.7, 3.55)	0.157
		Low	Ref.		Ref.		Ref.	
		Med	1.19 (-1.32, 3.69)	0.353	1.19 (-1.15, 3.52)	0.319	0.92 (-1.43. 3.28)	0.443
		High	-1.12 (-3.58, 1.35)	0.374	-0.67 (-3.01, 1.67)	0.573	-1.00 (-3.47, 1.47)	0.428
		p-trend		0.394		0.472		0.321
	PFOS	Cont.	-0.06 (-0.32, 0.20)	0.636	-0.08 (-0.34, 0.18)	0.542	-0.12 (-0.40, 0.15)	0.371
		Low	Ref.		Ref.		Ref.	
		Med	-0.01 (-2.55, 2.54)	0.996	0.52 (-1.87, 2.92)	0.668	0.31 (-2.10, 2.71)	0.802
		High	0.06 (-2.49, 2.60)	0.965	0.56 (-1.88, 2.99)	0.653	0.36 (-2.20, 2.91)	0.785
		p-trend		0.966		0.922		0.733
	**∑PFSAs**	Cont.	-0.06 (-0.31, 0.18)	0.598	-0.08 (-0.33, 0.16)	0.516	-0.12 (-0.38, 0.14)	0.351
		Low	Ref.		Ref.		Ref.	
		Med	0.12 (-2.42, 2.67)	0.926	0.74 (-1.67, 3.14)	0.548	0.50 (-1.93, 2.92)	0.688
		High	0.12 (-2.42, 2.67)	0.926	0.66 (-1.77, 3.09)	0.596	0.44 (-2.12, 3.01)	0.734
		p-trend		0.927		0.980		0.788
**PFCAs (ng/mL)**	PFOA	Cont.	-0.12 (-1.22, 0.97)	0.822	-0.12 (-1.25, 1.01)	0.835	-0.15 (-1.28, 0.98)	0.794
		Low	Ref.		Ref.		Ref.	
		Med	0.22 (-2.28, 2.72)	0.864	0.07 (-2.30, 2.45)	0.953	0.31 (-2.22, 2.85)	0.809
		High	-1.50 (-4.01, 1.02)	0.244	-0.72 (-3.14, 1.70)	0.560	-0.73 (-3.34, 1.89)	0.585
		p-trend		0.257		0.317		0.283
	PFNA	Cont.	-0.74 (-2.70, 1.22)	0.455	-0.83 (-2.79, 1.14)	0.406	-1.00 (-3.00, 1.00)	0.324
		Low	Ref.		Ref.		Ref.	
		Med	-0.68 (-3.20, 1.85)	0.599	-1.11 (-3.46, 1.25)	0.358	-1.22 (-3.58, 1.15)	0.314
		High	-0.26 (-2.78, 2.27)	0.842	0.38 (-1.99, 2.74)	0.755	0.23 (-2.19, 2.64)	0.853
		p-trend		0.837		0.820		0.670
	PFDA	Cont.	-0.36 (-2.59, 1.87)	0.752	-0.49 (-2.72, 1.75)	0.667	-0.69 (-2.95, 1.58)	0.548
		Low	Ref.		Ref.		Ref.	
		Med	0.11 (-2.44, 2.65)	0.935	0.52 (-1.84, 2.87)	0.668	0.30 (-2.09, 2.68)	0.808
		High	0.35 (-2.16, 2.85)	0.787	0.64 (-1.73, 3.00)	0.599	0.41 (-2.01, 2.83)	0.740
		p-trend		0.790		0.943		0.878
	PFUnA	Cont.	0.04 (-0.64, 0.72)	0.900	0.02 (-0.66, 0.70)	0.949	-0.02 (-0.71, 0.66)	0.947
		Low	Ref.		Ref.		Ref.	
		Med	-0.05 (-2.61, 2.51)	0.971	-0.02 (-2.40, 2.36)	0.986	-0.25 (-2.68, 2.18)	0.839
		High	0.91 (-1.57, 3.39)	0.472	0.42 (-1.90, 2.74)	0.723	0.12 (-2.23, 2.47)	0.922
		p-trend		0.479		0.610		0.764
	**∑PFCAs**	Cont.	0.04 (-0.27, 0.35)	0.805	0.02 (-0.29, 0.33)	0.902	-0.01 (-0.31, 0.31)	0.976
		Low	Ref.		Ref.		Ref.	
		Med	-1.68 (-4.20, 0.84)	0.191	-1.32 (-3.71, 1.07)	0.279	-1.64 (-4.07, 0.80)	0.187
		High	-0.32 (-2.80, 2.16)	0.802	-0.34 (-2.67, 1.99)	0.775	-0.66 (-3.02, 1.69)	0.582
		p-trend		0.798		0.678		0.516
**PFASs (ng/mL)**	**∑PFASs**	Cont.	-0.02 (-0.17, 0.14)	0.834	-0.03 (-0.18, 0.13)	0.731	-0.05 (-0.20, 0.11)	0.561
		Low	Ref.		Ref.		Ref.	
		Med	-0.26 (-2.82, 2.31)	0.844	0.58 (-1.82, 2.97)	0.635	0.32 (-2.08, 2.73)	0.792
		High	0.11 (-2.42, 2.63)	0.935	0.47 (-1.91, 2.86)	0.697	0.20 (-2.28, 2.69)	0.872
		p-trend		0.932		0.977		0.770
**Metals (µg/L)**	Hg	Cont.	-0.11 (-0.25, 0.02)	**0.093****	-0.12 (-0.25, 0.01)	**0.066****	-0.14 (-0.28, -0.01)	**0.040***
		Low	Ref.		Ref.		Ref.	
		Med	-0.70 (-3.20, 1.80)	0.584	-0.44 (-2.79, 1.91)	0.712	-0.57 (-2.99, 1.85)	0.645
		High	-0.70 (-3.24, 1.84)	0.589	-0.36 (-2.77, 2.04)	0.767	-0.47 (-2.96, 2.01)	0.709
		p-trend		0.589		0.810		0.688
	Pb	Cont.	-0.04 (-0.18, 0.10)	0.533	-0.03 (-0.17, 0.11)	0.702	-0.03 (-0.17, 0.11)	0.695
		Low	Ref.		Ref.		Ref.	
		Med	-0.74 (-3.24, 1.76)	0.560	-0.04 (-2.43, 2.35)	0.973	0.27 (-2.16, 2.71)	0.826

	High	0.18 (-2.40, 2.76)	0.891	0.48 (-1.94, 2.90)	0.697	0.60 (-1.86, 3.05)	0.635
	p-trend		0.898		0.958		0.937
Cd	Cont.	-0.58 (-2.17, 1.00)	0.467	-1.20 (-3.01, 0.61)	0.191	-1.20 (-3.02, 0.62)	0.192
	Low	Ref.		Ref.		Ref.	
	Med	1.243 (-1.25, 3.74)	0.328	1.12 (-1.22, 3.46)	0.349	1.35 (-0.99, 3.69)	0.258
	High	-1.29 (-3.78, 1.20)	0.311	-1.96 (-4.45, 0.53)	0.123	-2.05 (-4.59, 0.48)	0.112
	p-trend		0.319		0.121		0.108
Se	Cont.	-0.01 (-0.02, 0.00)	**0.050***	-0.01 (-0.02, 0.00)	**0.044***	-0.01 (-0.02, -0.00)	**0.028***
	Low	Ref.		Ref.		Ref.	
	Med	-0.27 (-2.74, 2.20)	0.828	0.25 (-2.10, 2.61)	0.832	0.02 (-2.38, 2.41)	0.990
	High	-1.48 (-4.03, 1.07)	0.255	-0.69 (-3.08, 1.71)	0.574	-0.89 (-3.31, 1.54)	0.474
	p-trend		0.270		0.362		0.286

n: Number of participants in parameter, β: Linear regression coefficient in score points, CI: Confidence interval, * Significant (p ≤ 0.050), * * Borderline significant (p ≤ 0.100), Adjustment model 1: Maternal plasma cotinine, maternal educational level, maternal age at delivery, Adjustment model 2: Adjustment model 1 + breast-feeding duration, Med = Medium, Cont. = Continuous, PCB: polychlorinated biphenyl, OCPs: Organochlorine pesticides, , PFSA: Perfluoroalkylated substances, PFCA: perfluorinated carboxylic acid, PFAS: Perfluoroalkylated Substances

Table S5: Linear regression analysis of associations between multiple prenatal exposures and continuous SDQ score: Greenlandic children 3-5 years of age born 2014-2016 in the ACCEPT birth cohort.

	Unadjusted (n=95)		Adjustment model 1 (n=94)		Adjustment model 2 (n=93)	
	**β (95% CI)**	**p-Value**	**β (95% CI)**	**p-Value**	**β (95% CI)**	**p-Value**
∑PCB ⅄	-0.02 (-0.04, 0.00)	**0.061****	-0.02 (-0.04, 0.00)	**0.075****	-0.02 (-0.04, 0.00)	**0.074****
∑OCP ⅄	0.02 (0.01, 0.03)	**0.009***	0.02 (0.00, 0.03)	**0.010***	0.02 (0.01, 0.03)	**0.007***
∑PFSA ✦	0.04 (-0.34, 0.42)	0.832	0.03 (-0.35, 0.41)	0.858	0.06 (-0.32, 0.44)	0.758
∑PFCA ✦	-0.17 (-0.56, 0.23)	0.406	-0.18 (-0.57, 0.21)	0.358	-0.28 (-0.68, 0.13)	0.180
Hg ★	-0.03 (-0.27, 0.22)	0.817	-0.05 (-0.29, 0.20)	0.713	-0.06 (-0.31, 0.18)	0.613
Pb ★	-0.02 (-0.16, 0.13)	0.829	0.00 (-0.14, 0.14)	0.979	0.00 (-0.14, 0.14)	0.984
Cd ★	-0.43 (-1.99, 1.14)	0.590	-0.87 (-2.65, 0.92)	0.339	-0.90 (-2.67, 0.88)	0.318
Se ★	-0.01 (-0.03, 0.01)	0.300	-0.01 (-0.02, 0.01)	0.358	-0.01 (-0.02, 0.01)	0.370

All exposures were included in one model to adjust for possible confounding by simultaneous exposure. Adjustment model 1: Maternal plasma cotinine, maternal educational level, maternal age at delivery, Adjustment model 2: Adjustment model 1 + breast-feeding duration. n: Number of participants in parameter, β: Linear regression coefficient in score points, CI: Confidence interval, * Significant (p ≤ 0.050), * * Borderline significant (p ≤ 0.100), PCB: polychlorinated biphenyl´, OCPs: Organochlorine pesticides, , PFSA: Perfluoroalkylated substances, PFCA: perfluorinated carboxylic acid, ⅄ (µg/kg lipid), ✦ (ng/mL), ★ (µg/L).

Table S6: Linear regression analysis of the associations between prenatal POP and metal exposure and SDQ score.

	Boys (n=51)				Girls (n=44)			
	Unadjusted		Adjustment model 1		Unadjusted		Adjustment model 1	
	β (95% CI)	p-Value	β (95% CI)	p-Value	β (95% CI)	p-Value	β (95% CI)	p-Value
PCB118 ⅄	0.10 (-0.10, 0.29)	0.320	0.13 (-0.10, 0.35)	0.273	-0.08 (-0.32, 0.16)	0.499	-0.05 (-0.29, 0.20)	0.687
PCB138 ⅄	0.02 (-0.05, 0.10)	0.521	0.02 (-0.06, 0.11)	0.583	-0.01 (-0.09, 0.07)	0.761	-0.00 (-0.09, 0.08)	0.921
PCB153 ⅄	0.01 (-0.02, 0.04)	0.602	0.01 (-0.03, 0.04)	0.733	-0.01 (-0.05, 0.02)	0.502	-0.01 (-0.04, 0.03)	0.672
PCB156 ⅄	-0.11 (-0.66, 0.44)	0.679	-0.12 (-0.69, 0.45)	0.671	-0.20 (-0.69, 0.30)	0.426	-0.08 (-0.60, 0.45)	0.771
PCB170 ⅄	-0.01 (-0.22, 0.20)	0.930	-0.02 (-0.24, 0.20)	0.879	-0.10 (-0.29, 0.10)	0.334	-0.06 (-0.26, 0.15)	0.585
PCB180 ⅄	0.00 (-0.06, 0.07)	0.939	-0.00 (-0.07, 0.07)	0.959	-0.03 (-0.10, 0.03)	0.325	-0.02 (-0.09, 0.05)	0.552
PCB183 ⅄	0.08 (-0.56, 0.71)	0.808	0.07 (-0.67, 0.80)	0.860	-0.29 (-0.96, 0.38)	0.392	-0.17 (-0.87, 0.54)	0.635
PCB187 ⅄	0.03 (-0.12 0.18)	0.692	0.03 (-0.14, 0.20)	0.717	-0.08 (-0.23, 0.08)	0.329	-0.05 (-0.21, 0.11)	0.514
PCB99 ⅄	0.09 (-0.11, 0.28)	0.383	0.10 (-0.13, 0.33)	0.389	0.02 (-0.22, 0.27)	0.854	0.03 (-0.22, 0.29)	0.789
**∑PCBs** ⅄	0.00 (-0.01, 0.01)	0.650	0.00 (-0.01, 0.02)	0.742	-0.01 (-0.02, 0.01)	0.347	-0.00 (-0.02, 0.01)	0.550
cis-Nona-chlor ⅄	0.09 (-0.14, 0.31)	0.448	0.012 (-0.13, 0.36)	0.350	-0.01 (-0.27, 0.25)	0.947	0.01 (-0.26, 0.28)	0.953
Hexachlorobenzene ⅄	0.03 (-0.05, 0.10)	0.474	0.03 (-0.06, 0.12)	0.469	-0.00 (-0.09, 0.08)	0.985	-0.00 (-0.09, 0.08)	0.966
Mirex ⅄	0.07 (-0.53, 0.66)	0.828	0.02 (-0.64, 0.69)	0.943	-0.33 (-0.88, 0.22)	0.235	-0.29 (-0.84, 0.26)	0.296
Oxychlordane ⅄	0.04 (-0.05, 0.12)	0.378	0.04 (-0.05, 0.13)	0.400	0.01 (-0.09, 0.10)	0.888	0.01 (-0.09, 0.10)	0.870
p,p'-DDE ⅄	0.01 (-0.00, 0.03)	0.168	0.01 (-0.01, 0.03)	0.164	-0.00 (-0.02, 0.01)	0.761	-0.00 (-0.02, 0.01)	0.800
ß-HCH ⅄	0.25 (-0.28, 0.77)	0.355	0.32 (-0.28, 0.92)	0.292	0.04 (-0.64, 0.72)	0.903	0.19 (-0.52, 0.89)	0.600
transNonachlor ⅄	0.02 (-0.02, 0.05)	0.401	0.02 (-0.02, 0.06)	0.330	0.00 (-0.04, 0.05)	0.899	0.01 (-0.04, 0.05)	0.798
**∑OCPs** ⅄	0.01 (-0.00, 0.01)	0.256	0.01 (-0.00, 0.01)	0.238	-0.00 (-0.01, 0.01)	0.881	0.00 (-0.01, 0.01)	0.931
**∑Legacy-POPs** ⅄	0.00 (-0.00, 0.01)	0.379	0.00 (-0.00, 0.01)	0.391	-0.00 (-0.01, 0.00)	0.621	-0.00 (-0.01, 0.01)	0.758
**∑Lip-POPs** ⅄	0.00 (-0.00, 0.01)	0.393	0.00 (-0.00, 0.01)	0.306	-0.00 (-0.01, 0.00)	0.627	-0.00 (-0.01, 0.01)	0.761
PFHxS ✦	-3.43 (-10.7, 3.83)	0.347	-3.58 (-11.4, 4.26)	0.363	-2.98 (-10.2, 4.26)	0.410	-2.50 (-9.99, 4.99)	0.504
PFHpS ✦	-3.58 (-20.7, 13.5)	0.675	-4.50 (-22.3, 13.3)	0.612	-10.3 (-27.4, 6.72)	0.228	-9.56 (-27.0, 7.91)	0.275
PFOS ✦	-0.04 (-0.41, 0.33)	0.825	-0.06 (-0.44, 0.33)	0.763	-0.10 (-0.48, 0.28)	0.605	-0.12 (-0.51, 0.27)	0.530
**∑PFSAs** ✦	-0.05 (-0.39, 0.30)	0.794	-0.06 (-0.43, 0.30)	0.737	-0.10 (-0.46, 0.26)	0.581	-0.12 (-0.48, 0.25)	0.519
PFOA ✦	0.03 (-1.54, 1.60)	0.974	0.16 (-1.57, 1.89)	0.855	-0.33 (-1.91, 1.25)	0.674	-0.53 (-2.15, 1.09)	0.512
PFNA ✦	-0.39 (-3.07, 2.29)	0.771	-0.49 (-3.27, 2.29)	0.724	-1.50 (-4.66, 1.66)	0.343	-1.54 (-4.80, 1.72)	0.345
PFDA ✦	-0.52 (-3.73, 2.68)	0.745	-0.48 (-3.92, 2.97)	0.782	-0.17 (-3.44, 3.11)	0.919	-0.72 (-4.08, 2.65)	0.669
PFUnA ✦	-0.14 (-1.06, 0.79)	0.769	-0.08 (-1.06, 0.91)	0.878	0.37 (-0.71, 1.45)	0.494	0.14 (-0.99, 1.26)	0.811
**∑PFCAs** ✦	-0.06 (-0.50, 0.39)	0.801	-0.02 (-0.48, 0.44)	0.933	0.17 (-0.28, 0.61)	0.455	0.05 (-0.43, 0.52)	0.846
**∑PFAS'** ✦	-0.03 (-0.24, 0.18)	0.778	-0.03 (-0.25, 0.20)	0.805	0.00 (-0.23, 0.24)	0.975	-0.04 (-0.28, 0.20)	0.747
Hg ★	-0.11 (-0.27, 0.05)	0.170	-0.12 (-0.28, 0.05)	0.156	-0.13 (-0.41, 0.16)	0.373	-0.16 (-0.45, 0.13)	0.270
Pb★	-0.01 (-0.26, 0.24)	0.938	-0.01 (-0.27, 0.25)	0.950	-0.06 (-0.23, 0.10)	0.436	-0.04 (-0.21, 0.13)	0.663
Cd ★	-0.85 (-3.48, 1.79)	0.523	-2.19 (-5.40, 1.01)	0.174	-0.38 (-2.39, 1.63)	0.704	-0.51 (-2.80, 1.79)	0.658
Se ★	-0.01 (-0.02, 0.00)	**0.075****	-0.01 (-0.02, 0.00)	**0.069****	-0.01 (-0.03, 0.02)	0.553	-0.01 (-0.03, 0.02)	0.632

n: Number of participants in parameter, β: Linear regression coefficient in score points, CI: Confidence interval, * Significant (p ≤ 0.050), * * Borderline significant (p ≤ 0.100), Adjustment model 1: Maternal plasma cotinine, maternal educational level, maternal age at delivery, Med = Medium, Cont. = Continuous, ∑LipPOPs = ∑LipophilicPOPs, ⅄ (µg/kg lipid), ✦ (ng/mL), ★ (µg/L), PCB: polychlorinated biphenyl, OCPs: Organochlorine pesticides, , PFSA: Perfluoroalkylated substances, PFCA: perfluorinated carboxylic acid, PFAS: Perfluoroalkylated Substances

Table S7: Linear regression analysis of the associations between prenatal POP and metal exposure and continuous hyperactivity score: Greenlandic children 3-5 years of age born 2014-2016, the ACCEPT birth cohort.

All (n = 101)			Unadjusted		Adjustment model 1		Adjustment model 2	
			β (95% CI)	p-Value	β (95% CI)	p-Value	β (95% CI)	p-Value
**PCBs (µg/kg lipid)**	PCB118	Cont.	0.03 (-0.02, 0.08)	0.275	0.03 (-0.03, 0.08)	0.301	0.03 (-0.02, 0.09)	0.259
		Low	Ref.		Ref.		Ref.	
		Med	-0.17 (-1.07, 0.73)	0.712	-0.21 (-1.13, 0.71)	0.653	-0.20 (-1.14, 0.74)	0.680
		High	0.00 (-0.89, 0.89)	1.000	0.18 (-0.77, 1.13)	0.712	0.20 (-0.81, 1.20)	0.701
		p-trend		1.000		0.971		0.925
	PCB138	Cont.	0.01 (-0.01, 0.03)	0.400	0.01 (-0.01, 0.03)	0.485	0.01 (-0.01, 0.03)	0.441
		Low	Ref.		Ref.		Ref.	
		Med	-0.07 (-0.96, 0.82)	0.878	-0.18 (-1.09, 0.72)	0.690	-0.19 (-1.12, 0.74)	0.691
		High	0.11 (-0.78, 1.01)	0.806	0.24 (-0.69, 1.17)	0.616	0.24 (-0.72, 1.19)	0.629
		p-trend		0.813		0.916		0.843
	PCB153	Cont.	0.00 (-0.01, 0.01)	0.442	0.00 (-0.01, 0.01)	0.529	0.00 (-0.01, 0.01)	0.488
		Low	Ref.		Ref.		Ref.	
		Med	-0.59 (-1.46, 0.29)	0.189	-0.71 (+1.61, 0.19)	0.120	-0.75 (-1.67, 0.16)	0.106
		High	0.19 (-0.71, 1.08)	0.681	0.26 (-0.65, 1.17)	0.579	0.21 (-0.72, 1.14)	0.661
		p-trend		0.712		0.777		0.736
	PCB156	Cont.	0.04 (-0.09, 0.17)	0.560	0.03 (-0.10, 0.17)	0.617	0.04 (-0.10, 0.17)	0.592
		Low	Ref.		Ref.		Ref.	
		Med	0.53 (-0.37, 1.42)	0.248	0.45 (-0.49, 1.40)	0.350	0.47 (-0.49, 1.42)	0.338
		High	0.35 (-0.54, 1.25)	0.442	0.54 (-0.40, 1.48)	0.256	0.58 (-0.36, 1.53)	0.228
		p-trend		0.454		0.501		0.457
	PCB170	Cont.	0.01 (-0.04, 0.06)	0.634	0.01 (-0.04, 0.06)	0.705	0.01 (-0.04, 0.06)	0.682
		Low	Ref.		Ref.		Ref.	
		Med	0.03 (-0.86, 0.92)	0.949	0.14 (-0.81, 1.09)	0.768	0.13 (-0.83, 1.28)	0.497
		High	0.16 (-0.74, 1.06)	0.727	0.34 (-0.60, 1.28)	0.478	0.33 (-0.62, 1.28)	0.785
		p-trend		0.731		0.811		0.787
	PCB180	Cont.	0.00 (-0.01, 0.02)	0.611	0.00 (-0.01, 0.02)	0.685	0.00 (-0.01, 0.02)	0.659
		Low	Ref.		Ref.		Ref.	
		Med	0.07 (-0.84, 0.97)	0.886	0.03 (-0.92, 0.97)	0.957	0.02 (-0.94, 0.97)	0.971
		High	0.03 (-0.89, 0.95)	0.948	0.21 (-0.75, 1.17)	0.672	0.21 (-0.76, 1.18)	0.670
		p-trend		0.952		0.967		0.990
	PCB183	Cont.	0.05 (-0.11, 0.21)	0.527	0.04 (-0.13, 0.22)	0.624	0.05 (-0.13, 0.23)	0.578
		Low	Ref.		Ref.		Ref.	
		Med	-0.34 (-1.22, 0.54)	0.451	-0.40 (-1.33, 0.54)	0.406	-0.40 (-1.34, 0.54)	0.407
		High	0.26 (-0.64, 1.17)	0.571	0.33 (-0.64, 1.29)	0.504	0.33 (-0.64, 1.30)	0.503
		p-trend		0.586		0.676		0.647
	PCB187	Cont.	0.01 (-0.03, 0.0)	0.498	0.01 (-0.03, 0.05)	0.570	0.01 (-0.03, 0.05)	0.537
		Low	Ref.		Ref.		Ref.	
		Med	-0.62 (-1.51, 0.27)	0.171	-0.68 (-1.57, 0.21)	0.136	-0.69 (-1.59, 0.22)	0.139
		High	0.09 (-0.79, 0.97)	0.844	0.17 (-0.73, 1.06)	0.716	0.19 (-0.72, 1.09)	0.688
		p-trend		0.848		0.942		0.893
	PCB99	Cont.	0.03 (-0.02, 0.08)	0.235	0.03 (-0.03, 0.09)	0.280	0.03 (-0.02, 0.09)	0.250
		Low	Ref.		Ref.		Ref.	
		Med	0.47 (-0.44, 1.38)	0.313	0.18 (-0.75, 1.11)	0.699	0.21 (-0.74, 1.16)	0.659
		High	0.10 (-0.78, 0.98)	0.821	0.09 (-0.83, 1.01)	0.848	0.12 (-0.82, 1.05)	0.806
		p-trend		0.836		0.990		0.921
	**∑PCBs**	Cont.	0.00 (-0.00, 0.00)	0.445	0.00 (-0.00, 0.00)	0.528	0.00 (-0.00, 0.00)	0.485
		Low	Ref.		Ref.		Ref.	
		Med	-0.62 (-1.50, 0.26)	0.169	-0.75 (-1.66, 0.17)	0.110	-0.71 (-1.64, 0.22)	0.133
		High	0.11 (-0.78, 1.00)	0.813	0.19 (-0.75, 1.13)	0.693	0.20 (-0.75, 1.14)	0.684
		p-trend		0.829		0.939		0.891
	**∑LegacyPOPs**	Cont.	0.00 (-0.00, 0.00)	0.319	0.00 (-0.00, 0.00)	0.377	0.00 (-0.00, 0.00)	0.340
		Low	Ref.		Ref.		Ref.	
		Med	-0.38 (-1.27, 0.50)	0.398	-0.58 (-1.47, 0.30)	0.197	-0.57 (-1.48, 0.35)	0.225
		High	0.23 (-0.67, 1.12)	0.622	0.40 (-0.50, 1.30)	0.380	0.42 (-0.50, 1.34)	0.369
		p-trend		0.637		0.713		0.639
		Cont.	0.00 (-0.00, 0.00)	0.505	0.00 (-0.00, 0.00)	0.586	0.00 (-0.00, 0.00)	0.504

	**∑Lipophilic POPs**	Low	Ref.		Ref.		Ref.	
		Med	-0.38 (-1.28, 0.53)	0.414	-0.56 (-1.48, 0.35)	0.225	-0.59 (-1.52, 0.35)	0.221
		High	0.17 (-0.74, 1.09)	0.710	0.32 (-0.59, 1.23)	0.487	0.36 (-0.56, 1.28)	0.440
		p-trend		0.724		0.760		0.661
**PFSAs (ng/mL)**	PFHxS	Cont.	0.03 (-1.76, 1.82)	0.972	-0.08 (-1.97, 1.82)	0.937	0.05 (-1.98, 2.09)	0.959
		Low	Ref.		Ref.		Ref.	
		Med	0.09 (-0.80, 0.98)	0.846	0.09 (-0.79, 0.97)	0.845	0.13 (-0.76, 1.03)	0.769
		High	-0.17 (-1.07, 0.73)	0.711	-0.05 (-0.96, 0.86)	0.918	0.08 (-0.88, 1.04)	0.869
		p-trend		0.717		0.613		0.668
	PFHpS	Cont.	-0.24 (-4.50, 4.02)	0.910	-0.49 (-4.91, 3.94)	0.828	-0.12 (-4.88, 4.65)	0.962
		Low	Ref.		Ref.		Ref.	
		Med	0.15 (-0.75, 1.04)	0.747	0.09 (-0.78, 0.96)	0.837	0.15 (-0.74, 1.03)	0.742
		High	0.04 (-0.86, 0.94)	0.932	0.11 (-0.79, 1.01)	0.808	0.27 (-0.69, 1.23)	0.581
		p-trend		0.931		0.975		0.893
	PFOS	Cont.	-0.01 (-0.10, 0.08)	0.872	-0.01 (-0.11, 0.08)	0.819	-0.00 (-0.11, 0.10)	0.949
		Low	Ref.		Ref.		Ref.	
		Med	-0.68 (-1.56, 0.21)	0.133	-0.58 (-1.45, 0.29)	0.190	-0.64 (-1.52, 0.23)	0.151
		High	-0.19 (-1.08, 0.70)	0.672	0.08 (-0.83, 0.98)	0.871	0.22 (-0.74, 1.17)	0.653
		p-trend		0.670		0.585		0.680
	**∑PFSAs**	Cont.	-0.01 (-0.09, 0.08)	0.879	-0.01 (-0.10, 0.08)	0.823	-0.00 (-0.10, 0.09)	0.953
		Low	Ref.		Ref.		Ref.	
		Med	-0.68 (-1.56, 0.21)	0.133	-0.60 (-1.47, 0.28)	0.181	-0.67 (-1.55, 0.21)	0.136
		High	-0.19 (-1.08, 0.70)	0.672	0.07 (-0.83, 0.97)	0.880	0.20 (-0.75, 1.16)	0.677
		p-trend		0.670		0.585		0.683
**PFCAs (ng/mL)**	PFOA	Cont.	0.16 (-0.24, 0.55)	0.426	0.17 (-0.25, 0.59)	0.419	0.17 (-0.25, 0.60)	0.416
		Low	Ref.		Ref.		Ref.	
		Med	0.24 (-0.65, 1.13)	0.604	0.15 (-0.74, 1.04)	0.741	0.34 (-0.62, 1.27)	0.502
		High	-0.23 (-1.13, 0.66)	0.612	-0.24 (-1.14, 0.66)	0.605	-0.04 (-1.02, 0.94)	0.930
		p-trend		0.624		0.570		0.611
	PFNA	Cont.	0.07 (-0.63, 0.78)	0.838	0.05 (-.0.68, 0.78)	0.885	0.10 (-0.66, 0.85)	0.803
		Low	Ref.		Ref.		Ref.	
		Med	-0.50 (-1.38, 0.38)	0.265	-0.62 (-1.48, 0.23)	0.152	-0.64 (-1.51, 0.22)	0.145
		High	0.09 (-0.81, 0.99)	0.838	0.31 (-0.57, 1.19)	0.494	0.40 (-0.50, 1.30)	0.383
		p-trend		0.861		0.922		0.833
	PFDA	Cont.	-0.02 (-0.83, 0.79)	0.960	-0.02 (-0.85, 0.81)	0.966	0.02 (-0.83, 0.87)	0.959
		Low	Ref.		Ref.		Ref.	
		Med	-0.50 (-1.39, 0.39)	0.270	-0.53 (-1.40, 0.34)	0.236	-0.62 (-1.51, 0.27)	0.175
		High	-0.19 (-1.09, 0.70)	0.671	0.04 (-0.85, 0.92)	0.939	0.08 (-0.83, 0.99)	0.860
		p-trend		0.670		0.720		0.809
	PFUnA	Cont.	-0.12 (-0.36, 0.13)	0.350	-0.11 (-0.36, 0.14)	0.378	-0.11 (-0.36, 0.15)	0.413
		Low	Ref.		Ref.		Ref.	
		Med	-0.32 (-1.21, 0.58)	0.491	-0.32 (-1.19, 0.56)	0.479	-0.31 (-1.21, 0.59)	0.509
		High	-0.38 (-1.27, 0.51)	0.400	-0.35 (-1.23, 0.52)	0.430	-0.38 (-1.28, 0.51)	0.403
		p-trend		0.407		0.469		0.531
	**∑PFCAs**	Cont.	-0.03 (-0.14, 0.09)	0.646	-0.02 (-0.14, 0.09)	0.684	-0.02 (-0.14, 0.10)	0.733
		Low	Ref.		Ref.		Ref.	
		Med	-1.15 (-2.03, -0.28)	**0.009***	-1.07 (-1.94, -0.21)	**0.015***	-1.14 (-2.03, -0.25)	**0.012***
		High	-0.53 (-1.40, 0.34)	0.231	-0.52 (-1.38, 0.34)	0.236	-0.51 (-1.39, 0.36)	0.252
		p-trend		0.250		0.265		0.310
**PFASs (ng/mL)**	**∑PFAS'**	Cont.	-0.01 (-0.06, 0.05)	0.748	-0.01 (-0.07, 0.05)	0.734	-0.01 (-0.07, 0.05)	0.835
		Low	Ref.		Ref.		Ref.	
		Med	-0.71 (-1.60, 0.18)	0.120	-0.54 (-1.42, 0.34)	0.232	-0.55 (-1.43, 0.34)	0.226
		High	-0.35 (-1.24, 0.53)	0.434	-0.11 (-1.00, 0.78)	0.810	-0.03 (-0.96, 0.91)	0.956
		p-trend		0.444		0.466		0.550
**Metals (μg/L)**	Hg	Cont.	-0.03 (-0.08, 0.02)	0.245	-0.03 (-0.08, 0.02)	0.272	-0.03 (-0.08, 0.03)	0.316
		Low	Ref.		Ref.		Ref.	
		Med	-0.23 (-1.13, 0.67)	0.612	-0.17 (-1.07, 0.72)	0.703	-0.08 (-1.00, 0.85)	0.872
		High	-0.25 (-1.14, 0.64)	0.581	-0.13 (-1.01, 0.74)	0.766	-0.07 (-0.97, 0.84)	0.889
		p-trend		0.584		0.630		0.670
	Pb	Cont.	-0.00 (-0.05, 0.05)	0.937	-0.00 (-0.06, 0.05)	0.893	-0.00 (-0.06, 0.05)	0.906
		Low	Ref.		Ref.		Ref.	
		Med	-0.38 (-1.27, 0.51)	0.400	-0.27 (-1.16, 0.62)	0.547	-0.24 (-1.15, 0.67)	0.599
		High	-0.05 (-0.94, 0.85)	0.921	0.09 (-0.80, 0.98)	0.839	0.06 (-0.85, 0.97)	0.901

	p-trend		0.916		0.842		0.836
Cd	Cont.	-0.13 (-0.69, 0.43)	0.645	-0.27 (-0.94, 0.40)	0.431	-0.26 (-0.94, 0.42)	0.448
	Low	Ref.		Ref.		Ref.	
	Med	0.04 (-0.85, 0.93)	0.930	-0.04 (-0.93, 0.86)	0.936	-0.02 (-0.92, 0.89)	0.973
	High	-0.56 (-1.46, 0.34)	0.221	-0.60 (-1.56, 0.37)	0.226	-0.58 (-1.58, 0.42)	0.258
	p-trend		0.225		0.137		0.151
Se	Cont.	-0.00 (-0.01, 0.00)	0.199	-0.00 (-0.01, 0.00)	0.224	-0.00 (-0.01, 0.00)	0.260
	Low	Ref.		Ref.		Ref.	
	Med	-0.24 (-1.13, 0.66)	0.605	-0.36 (-1.25, 0.54)	0.435	-0.33 (-1.25, 0.58)	0.475
	High	-0.20 (-1.10, 0.70)	0.668	-0.14 (-1.02, 0.75)	0.759	-0.14 (-1.04, 0.77)	0.763
	p-trend		0.670		0.712		0.745

n: Number of participants in parameter, β: Linear regression coefficient in score points, CI: Confidence interval, * Significant (p ≤ 0.050), * * Borderline significant (p ≤ 0.100), Adjustment model 1: Maternal plasma cotinine, maternal educational level, maternal age at delivery, Adjustment model 2: Adjustment model 1 + breast-feeding duration, Med = Medium, Cont. = Continuous, PCB: polychlorinated biphenyl, OCPs: Organochlorine pesticides, , PFSA: Perfluoroalkylated substances, PFCA: perfluorinated carboxylic acid, PFAS: Perfluoroalkylated Substances

Table S8: Linear regression analysis of associations between multiple prenatal exposures and continuous hyperactivity score: Greenlandic children 3-5 years of age born 2014-2016 in the ACCEPT birth cohort.

	Unadjusted (n=101)		Adjustment model 1 (n=100)		Adjustment model 2 (n=99)	
	**β (95% CI)**	**p-Value**	**β (95% CI)**	**p-Value**	**β (95% CI)**	**p-Value**
∑PCB ⅄	-0.00 (-0.01, 0.01)	0.557	-0.00 (-0.01, 0.01)	0.547	-0.00 (-0.01, 0.01)	0.561
∑OCP ⅄	0.00 (-0.00, 0.01)	0.134	0.00 (-0.00, 0.01)	0.148	0.00 (-0.00, 0.01)	0.158
∑PFSA ✦	-0.04 (-0.19, 0.10)	0.571	-0.04 (-0.19, 0.11)	0.617	-0.04 (-0.19, 0.12)	0.639
∑PFCA ✦	-0.03 (-0.18, 0.11)	0.655	-0.03 (-0.18, 0.12)	0.651	-0.03 (-0.19, 0.13)	0.688
Hg ⋆	-0.01 (-0.10, 0.08)	0.810	-0.01 (-0.10, 0.08)	0.819	-0.01 (-0.10, 0.08)	0.826
Pb ⋆	-0.00 (-0.05, 0.05)	0.974	0.00 (-0.06, 0.05)	0.990	0.00 (-0.06, 0.06)	0.988
Cd ⋆	-0.15 (-0.72, 0.43)	0.617	-0.19 (-0.88, 0.49)	0.576	-0.20 (-0.89, 0.50)	0.576
Se ⋆	-0.00 (-0.01, 0.00)	0.504	-0.00 (-0.01, 0.00)	0.535	-0.00 (-0.01, 0.00)	0.541

All exposures were included in one model to adjust for possible confounding by simultaneous exposure. Adjustment model 1: Maternal plasma cotinine, maternal educational level, maternal age at delivery, Adjustment model 2: Adjustment model 1 + breast-feeding duration. n: Number of participants in parameter, β: Linear regression coefficient in score points, CI: Confidence interval, * Significant (p ≤ 0.050), * * Borderline significant (p ≤ 0.100), PCB: polychlorinated biphenyl, OCPs: Organochlorine pesticides, , PFSA: Perfluoroalkylated substances, PFCA: perfluorinated carboxylic acid, PFAS: Perfluoroalkylated Substances, ⅄ (µg/kg lipid), ✦ (ng/mL), ★ (µg/L).

Table S9: Linear regression analysis of the associations between prenatal POP and metal exposure and hyperactivity score.

	Boys (n=55)				Girls (n=46)			
	Unadjusted		Adjustment model 1		Unadjusted		Adjustment model 1	
	β (95% CI)	p-Value	β (95% CI)	p-Value	β (95% CI)	p-Value	β (95% CI)	p-Value
PCB118 ⅄	0.03 (-0.03, 0.09)	0.312	0.04 (-0.03, 0.11)	0.278	0.00 (-0.09, 0.10)	0.931	0.01 (-0.10, 0.11)	0.917
PCB138 ⅄	0.01 (-0.02, 0.03)	0.650	0.01 (-0.02, 0.03)	0.687	0.01 (-0.02, 0.04)	0.568	0.01 (-0.02, 0.04)	0.580
PCB153 ⅄	0.00 (-0.01, 0.01)	0.807	0.00 (-0.01, 0.01)	0.869	0.01 (-0.01, 0.02)	0.436	0.01 (-0.01, 0.02)	0.440
PCB156 ⅄	-0.03 (-0.21, 0.14)	0.725	-0.04 (-0.23, 0.14)	0.652	0.11 (-0.08, 0.30)	0.232	0.13 (-0.08, 0.33)	0.212
PCB170 ⅄	-0.01 (-0.07, 0.06)	0.831	-0.01 (-0.08, 0.06)	0.756	0.04 (-0.04, 0.11)	0.355	0.04 (-0.04, 0.12)	0.346
PCB180 ⅄	-0.00 (-0.02, 0.02)	0.830	-0.00 (-0.03, 0.02)	0.744	0.01 (-0.01, 0.04)	0.332	0.01 (-0.01, 0.04)	0.329
PCB183 ⅄	0.02 (-0.18, 0.21)	0.860	0.01 (-0.22, 0.25)	0.903	0.08 (-0.19, 0.35)	0.548	0.08 (-0.21, 0.37)	0.570
PCB187 ⅄	0.00 (-0.05, 0.05)	0.869	0.00 (-0.05, 0.06)	0.917	0.02 (-0.04, 0.08)	0.491	0.02 (-0.04, 0.09)	0.494
PCB99 ⅄	0.03 (-0.04, 0.09)	0.421	0.03 (-0.04, 0.10)	0.407	0.03 (-0.07, 0.12)	0.571	0.03 (-0.08, 0.13)	0.589
**∑PCBs** ⅄	0.00 (-0.00, 0.00)	0.834	0.00 (-0.00, 0.00)	0.889	0.00 (-0.00, 0.01)	0.447	0.00 (-0.00, 0.01)	0.449
cis-Nona-chlor ⅄	0.03 (-0.04, 0.10)	0.445	0.03 (-0.05, 0.11)	0.422	0.02 (-0.08, 0.12)	0.700	0.02 (-0.09, 0.13)	0.715
Hexachlorobenzene ⅄	0.00 (-0.02, 0.03)	0.737	0.01 (-0.02, 0.03)	0.690	-0.01 (-0.04, 0.03)	0.739	-0.01 (-0.04, 0.03)	0.735
Mirex ⅄	-0.04 (-0.23, 0.15)	0.640	-0.06 (-0.28, 0.16)	0.573	0.02 (-0.19, 0.23)	0.859	0.02 (-0.20, 0.24)	0.869
Oxychlordane ⅄	0.01 (-0.02, 0.04)	0.450	0.01 (-0.02, 0.04)	0.448	0.02 (-0.02, 0.05)	0.465	0.01 (-0.02, 0.05)	0.471
p,p'-DDE ⅄	0.00 (-0.00, 0.01)	0.357	0.00 (-0.00, 0.01)	0.360	0.00 (-0.01, 0.01)	0.629	0.00 (-0.01, 0.01)	0.661
ß-HCH ⅄	0.06 (-0.10 0.23)	0.457	0.08 (-0.11, 0.27)	0.423	0.08 (-0.19, 0.35)	0.571	0.09 (-0.21, 0.38)	0.544
transNonachlor ⅄	0.01 (-0.01, 0.02)	0.401	0.01 (-0.01, 0.02)	0.380	0.01 (-0.01, 0.02)	0.545	0.01 (-0.01, 0.03)	0.553
**∑OCPs** ⅄	0.00 (-0.00, 0.00)	0.411	0.00 (-0.00, 0.00)	0.400	0.00 (-0.00, 0.00)	0.651	0.00 (-0.00, 0.01)	0.674
**∑Legacy-POPs** ⅄	0.00 (-0.00, 0.00)	0.558	0.00 (-0.00, 0.00)	0.568	0.00 (-0.00, 0.00)	0.549	0.00 (-0.00, 0.00)	0.564
**∑Lip-POPs** ⅄	0.00 (-0.00, 0.00)	0.877	0.00 (-0.00, 0.00)	0.872	0.00 (-0.00, 0.00)	0.488	0.00 (-0.00, 0.00)	0.503
PFHxS ✦	-0.44 (-2.73, 1.85)	0.700	-0.59 (-3.11, 1.92)	0.638	0.30 (-2.62, 3.22)	0.837	0.32 (-2.81, 3.45)	0.838
PFHpS ✦	-2.40 (-7.80, 3.00)	0.376	-2.66 (-8.39, 3.07)	0.356	2.10 (-4.82, 9.02)	0.544	2.19 (-5.17, 9.54)	0.552
PFOS ✦	-0.05 (-0.16, 0.07)	0.409	-0.05 (-0.17, 0.07)	0.405	0.03 (-0.13, 0.18)	0.723	0.03 (-0.14, 0.19)	0.752
**∑PFSAs** ✦	-0.04 (-0.15, 0.06)	0.415	-0.05 (-0.16, 0.07)	0.409	0.03 (-0.12, 0.17)	0.722	0.02 (-0.13, 0.18)	0.750
PFOA ✦	0.03 (-0.48, 0.54)	0.899	-0.01 (-0.57, 0.56)	0.985	0.31 (-0.32, 0.93)	0.327	0.33 (-0.34, 0.99)	0.331
PFNA ✦	-0.22 (-1.08, 0.64)	0.607	-0.21 (-1.12, 0.69)	0.637	0.35 (-0.92, 1.63)	0.580	0.37 (-1.00, 1.73)	0.588
PFDA ✦	-0.46 (-1.49, 0.57)	0.374	-0.47 (-1.57, 0.65)	0.404	0.41 (-0.90, 1.72)	0.530	0.44 (-0.95, 1.84)	0.524
PFUnA ✦	-0.19 (-0.49, 0.10)	0.198	-0.19 (-0.51, 0.13)	0.227	-0.02 (-0.45, 0.42)	0.931	-0.02 (-0.49, 0.45)	0.929
**∑PFCAs** ✦	-0.08 (-0.22, 0.06)	0.277	-0.08 (-0.23, 0.07)	0.299	0.04 (-0.14, 0.21)	0.691	0.04 (-0.16, 0.24)	0.685
**∑PFAS'** ✦	-0.03 (-0.10, 0.03)	0.307	-0.04 (-0.11, 0.04)	0.312	0.02 (-0.07, 0.11)	0.663	0.02 (-0.08, 0.12)	0.681
Hg ★	-0.04 (-0.09, 0.01)	0.145	-0.04 (-0.09, 0.02)	0.188	-0.01 (-0.12, 0.11)	0.907	-0.00 (-0.13, 0.12)	0.955
Pb★	-0.00 (-0.08, 0.08)	0.923	-0.01 (-0.09, 0.08)	0.894	0.00 (-0.06, 0.07)	0.953	0.00 (-0.07, 0.07)	0.929
Cd ★	-0.34 (-1.13, 0.46)	0.403	-0.55 (-1.58, 0.47)	0.285	0.14 (-0.66, 0.94)	0.731	0.08 (-0.87, 1.03)	0.863
Se ★	-0.00 (-0.01, 0.00)	**0.087****	-0.00 (-0.01, 0.00)	0.112	0.00 (-0.01, 0.01)	0.980	0.00 (-0.01, 0.01)	0.931

n: Number of participants in parameter, β: Linear regression coefficient in score points, CI: Confidence interval, * Significant ($p \leq 0.050$), * * Borderline significant ($p \leq 0.100$), Adjustment model 1: Maternal plasma cotinine, maternal educational level, maternal age at delivery, Med = Medium, Cont. = Continuous, ∑LipPOPs = ∑LipophilicPOPs, ⅄(μg/kg lipid), ✦ (ng/mL), ★ (μg/L), PCB: polychlorinated biphenyl, OCPs: Organochlorine pesticides, , PFSA: Perfluoroalkylated substances, PFCA: perfluorinated carboxylic acid, PFAS: Perfluoroalkylated Substances

Table S10: Logistic regression analysis of associations between continuous prenatal POP and metal exposure and abnormal SDQ score: Greenlandic children 3-5 years of age born 2014-2016, the ACCEPT birth cohort.

		Unadjusted		Adjustment model 1		Adjustment model 2	
		**OR (95% CI)**	**p-Value**	**OR (95% CI)**	**p-Value**	**OR (95% CI)**	**p-Value**
**PCBs (µg/kg lipid)**	PCB118	1.06 (0.99, 1.14)	0.102	1.06 (0.97, 1.15)	0.215	1.05 (0.96, 1.15)	0.260
	PCB138	1.02 (0.99, 1.05)	0.189	1.01 (0.98, 1.05)	0.369	1.01 (0.98, 1.04)	0.446
	PCB153	1.01 (0.99, 1.02)	0.276	1.00 (0.99, 1.02)	0.518	1.00 (1.00, 1.029	0.606
	PCB156	0.95 (0.75, 1.21)	0.683	0.96 80.76, 1.22)	0.752	0.95 (0.74, 1.21)	0.667
	PCB170	1.00 (0.93, 1.09)	0.915	1.00 (0.92, 1.09)	1.000	1.00 (0.91, 1.09)	0.913
	PCB180	1.00 (0.98, 1.03)	0.720	1.00 (0.98, 1.03)	0.874	1.00 (0.97,1.03)	0.959
	PCB183	1.14 (0.90, 1.44)	0.284	1.11 (0.85, 1.45)	0.456	1.09 (0.83, 1.44)	0.547
	PCB187	1.03 (0.97, 1.09)	0.300	1.03 (0.97, 1.09)	0.412	1.02 (0.96, 1.09)	0.477
	PCB99	1.06 (0.99, 1.14)	0.115	1.05 (0.97, 1.15)	0.254	1.05 (0.96, 1.14)	0.317
	**∑PCBs**	1.00 (1.00, 1.01)	0.399	1.00 (1.00, 1.01)	0.635	1.00 (1.00, 1.01)	0.739
	**∑LegacyPOPs**	1.00 (1.00, 1.00)	0.107	1.00 (1.00, 1.00)	0.533	1.00 (1.00, 1.00)	0.270
	**∑LipophilicPOPs**	1.00 (1.00, 1.00)	0.128	1.00 (1.00, 1.00)	0.175	1.00 (1.00, 1.00)	0.211
**PFSAs (ng/mL)**	PFHxS	1.27 (0.07, 24.2)	0.874	1.01 (0.04, 26.0)	0.994	0.63 (0.02, 22.1)	0.799
	PFHpS	2.03 (0.00, 2109)	0.841	0.83 (0.00, 1395)	0.962	0.28 (0.00, 746)	0.750
	PFOS	1.09 (0.94, 1.26)	0.248	1.06 80.91, 1.24)	0.446	1.05 (0.87, 1.23)	0.599
	**∑PFSAs**	1.08 (0.94, 1.23)	0.271	1.06 (0.91, 1.22)	0.474	1.04 (0.89, 1.01)	0.633
**PFCAs (ng/mL)**	PFOA	0.81 (0.34, 1.98)	0.649	0.86 (0.36, 2.08)	0.739	0.85 (0.34, 2.11)	0.717
	PFNA	1.54 (0.53, 4.44)	0.426	1.35 (0.42, 4.28)	0.615	1.21 (0.37, 4.02)	0.753
	PFDA	1.82 (0.54, 6.12)	0.333	1.52 (0.42, 5.51)	0.522	1.41 (0.38, 5.19)	0.610
	PFUnA	1.15 (0.80, 1.65)	0.449	1.11 (0.76, 1.64)	0.582	1.12 (0.75, 1.66)	0.588
	**∑PFCAs**	1.06 (0.90, 1.25)	0.498	1.05 (0.88, 1.24)	0.618	1.04 (0.87, 1.25)	0.647
**PFASs (ng/mL)**	**∑PFAS'**	1.05 (0.96, 1.14)	0.305	1.03 (0.94, 1.13)	0.485	1.03 80.93, 1.13)	0.598
**Metals (µg/L)**	Hg	0.99 (0.90, 1.09)	0.848	0.98 (0.88, 1.09)	0.724	0.97 (0.88, 1.08)	0.609
	Pb	1.00 (0.93, 1.09)	0.947	1.00 (0.91, 1.11)	0.948	1.00 (0.91, 1.11)	0.974
	Cd	0.96 (0.37, 2.52)	0.942	0.63 (0.19, 2.10)	0.454	0.60 (0.17, 2.10)	0.421
	Se	1.00 (0.99, 1.01)	0.584	1.00 (0.99, 1.01)	0.504	1.00 (0.99, 1.01)	0.428

Normal/borderline n= 84, Abnormal n = 11, OR: Odds-ratio, CI: Confidence interval, * Significant (p ≤ 0.050), * * Borderline significant (p ≤ 0.100), Adjustment model 1: Maternal plasma cotinine, maternal educational level, maternal age at delivery, Adjustment model 2: Adjustment model 1 + breast-feeding duration, n ◊ = n (abnormal), ∑LipPOPs = ∑LipophilicPOPs, PCB: polychlorinated biphenyl, OCPs: Organochlorine pesticides, , PFSA: Perfluoroalkylated substances, PFCA: perfluorinated carboxylic acid, PFAS: Perfluoroalkylated Substances

Table S11: Logistic regression analysis of the associations between prenatal POP and metal exposure and SDQ score.

	Boys (normal/borderline n=44 / abnormal n=7)				Girls (normal/borderline n=40 / abnormal n=4)			
	Unadjusted		Adjustment model 1		Unadjusted		Adjustment model 1	
	OR (95% CI)	p-Value	OR (95% CI)	p-Value	OR (95% CI)	p-Value	OR (95% CI)	p-Value
PCB118 ⅄	1.08 (0.99, 1.18)	**0.074****	1.08 (0.96, 1.21)	0.208	0.98 (0.81, 1.17)	0.780	0.98 (0.83, 1.16)	0.845
PCB138 ⅄	1.03 (0.99, 1.06)	0.128	1.02 (0.98, 1.06)	0.382	1.00 (0.94, 1.06)	0.921	1.00 (0.95, 1.05)	0.941
PCB153 ⅄	1.01 (1.00, 1.02)	0.148	1.01 (0.99, 1.02)	0.456	1.00 (0.97, 1.02)	0.707	1.00 (0.97, 1.02)	0.771
PCB156 ⅄	1.02 (0.79, 1.33)	0.863	1.00 (0.74, 1.34)	0.989	0.78 (0.45, 1.36)	0.378	0.83 (0.48, 1.45)	0.516
PCB170 ⅄	1.04 (0.95, 1.14)	0.418	1.02 (0.92, 1.13)	0.688	0.89 (0.69, 1.14)	0.356	0.92 (0.73, 1.16)	0.462
PCB180 ⅄	1.02 (0.99, 1.04)	0.296	1.01 (0.98, 1.04)	0.584	0.97 (0.89, 1.05)	0.387	0.98 (0.91, 1.05)	0.488
PCB183 ⅄	1.22 (0.93, 1.60)	0.159	1.14 (0.81, 1.62)	0.455	0.91 (0.55, 1.53)	0.729	0.98 (0.60, 1.61)	0.933
PCB187 ⅄	1.05 (0.99, 1.13)	0.117	1.05 (0.97, 1.13)	0.274	0.96 (0.83, 1.10)	0.551	0.97 (0.85, 1.11)	0.660
PCB99 ⅄	1.06 (0.97, 1.15)	0.200	1.03 (0.92, 1.16)	0.568	1.06 (0.91, 1.23)	0.454	1.04 (0.90, 1.21)	0.584
**∑PCBs** ⅄	1.00 (1.00, 1.01)	0.182	1.00 (1.00, 1.01)	0.512	1.00 (0.96, 1.01)	0.519	1.00 (0.99, 1.01)	0.628
cis-Nona-chlor ⅄	1.10 (0.99, 1.21)	**0.073****	1.10 (0.97, 1.24)	0.128	1.04 (0.88, 1.23)	0.640	1.04 (0.88, 1.22)	0.685
Hexachlorobenzene ⅄	1.03 (0.99, 1.06)	0.110	1.02 (0.98, 1.06)	0.364	1.01 (0.96, 1.07)	0.670	1.01 (0.96, 1.06)	0.839
Mirex ⅄	1.25 (0.97, 1.63)	**0.091****	1.18 (0.86, 1.61)	0.316	0.88 (0.54, 1.43)	0.605	0.89 (0.54, 1.47)	0.658
Oxychlordane ⅄	1.03 (0.99, 1.07)	0.121	1.02 (0.98, 1.07)	0.303	1.01 (0.96, 1.07)	0.671	1.01 (0.95, 1.06)	0.818
p,p'-DDE ⅄	1.01 (1.00, 1.02)	**0.033***	1.01 (1.00, 1.02)	**0.074****	1.00 (0.99, 1.01)	0.690	1.00 (0.99, 1.01)	0.821
ß-HCH ⅄	1.19 (0.95, 1.48)	0.122	1.16 (0.97, 1.54)	0.327	0.97 (0.60, 1.58)	0.912	1.00 (0.64, 1.57)	0.985
transNonachlor ⅄	1.02 (1.00, 1.03)	**0.085****	1.01 (0.99, 1.03)	0.159	1.01 (0.98, 1.04)	0.623	1.01 (0.98, 1.04)	0.688
**∑OCPs** ⅄	1.00 (1.00, 1.00)	**0.047***	1.00 (1.00, 1.01)	0.115	1.00 (1.00, 1.01)	0.668	1.00 (1.00, 1.01)	0.793
**∑Legacy-POPs** ⅄	1.00 (1.00, 1.00)	**0.075****	1.00 (1.00, 1.01)	0.218	1.00 (1.00, 1.00)	0.978	1.00 (1.00, 1.00)	0.977
**∑Lip-POPs** ⅄	1.00 (1.00, 1.00)	**0.078****	1.00 (1.00, 1.01)	0.126	1.00 (1.00, 1.00)	0.935	1.00 (1.00, 1.00)	0.939
PFHxS ✦	2.06 (0.06, 76.6)	0.695	0.91 (0.01, 62.1)	0.965	0.32 (0.00, 95.1)	0.694	0.34 (0.00, 127)	0.721
PFHpS ✦	36.9 (0.01, 171625)	0.402	5.63 (0.00, 57110)	0.713	0.00 (0.00, 7340)	0.357	0.00 (0.00, 40045)	0.372
PFOS ✦	1.13 (0.95, 1.36)	0.168	1.10 (0.90, 1.35)	0.353	0.98 (0.74, 1.29)	0.871	0.96 (0.72, 1.28)	0.757
**∑PFSAs** ✦	1.12 (0.95, 1.33)	0.181	1.09 (0.90, 1.32)	0.378	0.98 (0.75, 1.27)	0.848	0.96 (0.73, 1.26)	0.744
PFOA ✦	1.09 (0.54, 2.19)	0.821	1.41 (0.66, 3.01)	0.372	0.08 (0.00, 1.80)	0.113	0.03 (0.00, 1.17)	**0.060****
PFNA ✦	2.19 (0.65, 7.33)	0.204	1.80 (0.47, 6.89)	0.392	0.30 (0.02, 5.05)	0.401	0.24 (0.01, 4.18)	0.327
PFDA ✦	3.24 (0.67, 15.6)	0.144	2.63 (0.44, 15.7)	0.288	0.41 (0.02, 8.28)	0.563	0.28 (0.01, 5.37)	0.397
PFUnA ✦	1.22 (0.81, 1.83)	0.344	1.18 (0.73, 1.92)	0.496	0.90 (0.40, 2.06)	0.808	0.79 (0.34, 1.83)	0.584
**∑PFCAs** ✦	1.13 (0.92, 1.39)	0.237	1.15 (0.91, 1.45)	0.249	0.88 (0.59, 1.33)	0.554	0.83 (0.55, 1.23)	0.344
**∑PFAS'** ✦	1.08 (0.97, 1.20)	0.165	1.07 (0.95, 1.21)	0.265	0.96 (0.81, 1.15)	0.666	0.93 (0.78, 1.12)	0.454
Hg ★	0.99 (0.88, 1.10)	0.810	0.96 (0.79, 1.16)	0.672	1.00 (0.81, 1.22)	0.976	0.97 (0.80, 1.18)	0.768
Pb★	1.01 (0.90, 1.14)	0.866	0.98 (0.85, 1.14)	0.804	1.00 (0.88, 1.13)	0.972	1.02 (0.88, 1.18)	0.806
Cd ★	0.82 (0.19, 3.52)	0.793	0.25 (0.03, 2.17)	0.208	1.21 (0.33, 4.42)	0.773	1.41 (0.29, 6.70)	0.670
Se ★	1.00 (0.99, 1.01)	0.544	1.00 (0.98, 1.01)	0.427	1.00 (0.98, 1.02)	0.931	1.00 (0.97, 1.02)	0.846

n: Number of participants in parameter, OR: Odds-ratio, CI: Confidence interval, * Significant (p ≤ 0.050), * * Borderline significant (p ≤ 0.100), Adjustment model 1: Maternal plasma cotinine, maternal educational level, maternal age at delivery, N/A: Test not applicable., ∑LipPOPs = ∑LipophilicPOPs, ⅄(µg/kg lipid), ✦ (ng/mL), ★ (µg/L), PCB: polychlorinated biphenyl, OCPs: Organochlorine pesticides, , PFSA: Perfluoroalkylated substances, PFCA: perfluorinated carboxylic acid, PFAS: Perfluoroalkylated Substances

Table S12: Logistic regression analysis of associations between continuous prenatal POP and metal exposure and abnormal hyperactivity score: Greenlandic children 3-5 years of age born 2014-2016, the ACCEPT birth cohort.

		Unadjusted		Adjustment model 1		Adjustment model 2	
		**OR (95% CI)**	**p-Value**	**OR (95% CI)**	**p-Value**	**OR (95% CI)**	**p-Value**
**PCBs (µg/kg lipid)**	PCB118	1.06 (0.97, 1.16)	0.176	1.10 (0.99, 1.23)	**0.085****	1.10 (0.97, 1.22)	**0.088****
	PCB138	1.02 (0.99, 1.06)	0.236	1.03 (0.99, 1.07)	0.126	1.03 (0.99, 1.07)	0.134
	PCB153	1.01 (0.99, 1.02)	0.206	1.01 (1.00, 1.03)	0.110	1.01 (1.00, 1.03)	0.119
	PCB156	1.12 (0.89, 1.42)	0.334	1.15 (0.90, 1.48)	0.263	1.15 (0.90, 1.46)	0.280
	PCB170	1.05 (0.96, 1.16)	0.269	1.07 (0.97, 1.18)	0.182	1.07 (0.97, 1.18)	0.196
	PCB180	1.02 (0.99, 1.05)	0.207	1.02 (0.99, 1.06)	0.132	1.02 (0.99, 1.06)	0.142
	PCB183	1.17 (0.85, 1.60)	0.336	1.30 (0.89, 1.88)	0.173	1.29 (0.89, 1.86)	0.176
	PCB187	1.05 (0.97, 1.12)	0.224	1.06 80.98, 1.15)	0.134	1.06 (0.98, 1.15)	0.142
	PCB99	1.06 (0.96, 1.16)	0.241	1.09 (0.98, 1.21)	0.125	1.09 (0.98, 1.21)	0.134
	**∑PCBs**	1.00 (1.00, 1.01)	0.186	1.01 (1.00, 1.01)	**0.096****	1.01 (1.00, 1.01)	0.104
	**∑Legacy-POPs**	1.00 (1.00, 1.00)	0.230	1.00 (1.00, 1.01)	0.133	1.00 (1.00, 1.01)	0.143
	**∑Lip-POPs**	1.00 (1.00, 1.00)	0.683	1.00 (1.00, 1.00)	0.469	1.00 (1.00, 1.00)	0.471
**PFSAs (ng/mL)**	PFHxS	9.80 (0.21, 462)	0.246	14.8 (0.28, 780)	0.183	23.6 (0.40, 1415)	0.130
	PFHpS	2.32 (0.00, 51169)	0.869	4.29 (0.00, 98784)	0.776	9.52 (0.00, 364275)	0.676
	PFOS	1.02 (0.82, 1.26)	0.888	1.03 (0.83, 1.28)	0.801	1.04 (0.83, 1.30)	0.718
	**∑PFSAs**	1.02 (0.83, 1.25)	0.847	1.03 (0.84, 1.26)	0.758	1.05 (0.85, 1.29)	0.673
**PFCAs (ng/mL)**	PFOA	0.09 (0.01, 1.31)	**0.078****	0.08 (0.01, 1.26)	**0.073****	0.09 (0.01, 1.53)	**0.094****
	PFNA	1.06 (0.20, 5.61)	0.942	1.16 (0.22, 6.02)	0.865	1.21 (0.23, 6.45)	0.822
	PFDA	1.09 (0.16, 7.27)	0.927	1.15 (0.17, 7.57)	0.888	1.18 (0.17, 8.12)	0.863
	PFUnA	0.92 (0.48, 1.75)	0.789	0.92 (0.47, 1.78)	0.793	0.92 (0.47, 1.78)	0.794
	**∑PFCAs**	0.86 (0.60, 1.23)	0.416	0.87 (0.61, 1.23)	0.414	0.86 (0.61, 1.23)	0.412
**PFASs (ng/mL)**	**∑PFAS'**	0.98 (0.86, 1.12)	0.778	0.99 (0.86, 1.13)	0.826	0.99 (0.86, 1.13)	0.867
**Metals (µg/L)**	Hg	0.98 (0.83, 1.16)	0.835	0.98 (0.84, 1.15)	0.984	0.99 (0.83, 1.17)	0.863
	Pb	0.99 (0.87, 1.13)	0.934	1.00 (0.88, 1.13)	0.991	1.00 (0.88, 1.13)	0.997
	Cd	0.65 (0.12, 3.47)	0.610	0.85 (0.12, 6.31)	0.875	0.93 (0.12, 6.93)	0.942
	Se	1.00 (1.00, 1.01)	0.479	1.00 (1.00, 1.01)	0.480	1.00 (1.00, 1.01)	0.389

Normal/borderline n= 96, Abnormal n = 5, OR: Odds-ratio, CI: Confidence interval, * Significant (p ≤ 0.050), * * Borderline significant (p ≤ 0.100), Adjustment model 1: Maternal plasma cotinine, maternal educational level, maternal age at delivery, Adjustment model 2: Adjustment model 1 + breast-feeding duration,, ∑LipPOPs = ∑LipophilicPOPs,

Table S13: Logistic regression analysis of the associations between prenatal POP and metal exposure and hyperactivity score.

	Boys (normal/borderline n= 51 / abnormal n =4)				Girls (normal/borderline n= 45 / abnormal n =1)			
	Unadjusted		Adjustment model 1		Unadjusted		Adjustment model 1	
	OR (95% CI)	p-Value	OR (95% CI)	p-Value	OR (95% CI)	p-Value	OR (95% CI)	p-Value
PCB118 ⅄	1.06 (0.96, 1.16)	0.243	1.13 (0.97, 1.30)	0.109	1.02 (0.75, 1.40)	0.894	1.02 (0.77, 1.35)	0.907
PCB138 ⅄	1.02 (0.98, 1.06)	0.284	1.04 (0.99, 1.09)	0.146	1.01 (0.92, 1.11)	0.847	1.01 (0.92, 1.11)	0.832
PCB153 ⅄	1.01 (0.99, 1.02)	0.384	1.01 (0.99, 1.03)	0.219	1.02 (0.98, 1.06)	0.359	1.02 (0.98, 1.06)	0.444
PCB156 ⅄	1.03 (0.74, 1.44)	0.844	1.04 (0.74, 1.45)	0.828	1.39 (0.89, 2.16)	0.147	3.15 (0.17, 57.9)	0.441
PCB170 ⅄	1.03 (0.91, 1.15)	0.658	1.04 (0.92, 1.17)	0.569	1.14 (0.95, 1.37)	0.172	1.26 (0.86, 1.85)	0.239
PCB180 ⅄	1.01 (0.97, 1.05)	0.669	1.01 (0.97, 1.05)	0.557	1.06 (0.99, 1.13)	0.129	1.15 (0.72, 1.83)	0.554
PCB183 ⅄	1.13 (0.81, 1.59)	0.474	1.26 (0.84, 1.91)	0.264	1.24 (0.54, 2.81)	0.612	1.25 (0.52, 3.04)	0.620
PCB187 ⅄	1.02 (0.94, 1.12)	0.623	1.04 (0.94, 1.14)	0.461	1.14 (0.94, 1.37)	0.179	1.23 (0.83, 1.81)	0.299
PCB99 ⅄	1.05 (0.96, 1.16)	0.305	1.10 (0.97, 1.25)	0.158	1.01 (0.73, 1.40)	0.959	1.04 (0.76, 1.41)	0.819
**∑PCBs** ⅄	1.00 (1.00, 1.01)	0.400	1.00 (1.00, 1.01)	0.239	1.01 (0.99, 1.02)	0.296	1.01 (0.99, 1.02)	0.360
cis-Nona-chlor ⅄	1.06 (0.94, 1.19)	0.360	1.08 (0.95, 1.23)	0.261	1.06 (0.77, 1.45)	0.725	1.11 (0.76, 1.61)	0.591
Hexachlorobenzene⅄	1.01 (0.97, 1.05)	0.523	1.03 (0.98, 1.08)	0.284	1.01 (0.91, 1.12)	0.847	1.02 (0.92, 1.12)	0.771
Mirex ⅄	1.05 (0.73, 1.51)	0.783	1.13 (0.74, 1.74)	0.561	2.58 (0.49, 13.7)	0.266	2.6E25 (0.00, -)	0.980
Oxychlordane ⅄	1.03 (0.99, 1.07)	0.208	1.04 (0.99, 1.09)	0.124	1.04 (0.95, 1.14)	0.398	1.04 (0.94, 1.16)	0.459
p,p'-DDE ⅄	1.00 (0.99, 1.01)	0.553	1.00 (1.00, 1.01)	0.385	1.00 (0.98, 1.02)	0.894	1.00 (0.99, 1.02)	0.883
ß-HCH ⅄	1.13 (0.87, 1.47)	0.373	1.26 (0.88, 1.80)	0.205	0.73 (0.21, 2.56)	0.619	0.84 (0.25, 2.88)	0.782
transNonachlor ⅄	1.01 (0.99, 1.03)	0.251	1.02 (0.99, 1.04)	0.173	1.02 (0.97, 1.08)	0.421	1.03 (0.97, 1.10)	0.363
**∑OCPs** ⅄	1.00 (1.00, 1.01)	0.415	1.00 (1.00, 1.01)	0.266	1.00 (0.99, 1.01)	0.690	1.00 (0.99, 1.01)	0.693
**∑Legacy-POPs** ⅄	1.00 (1.00, 1.00)	0.396	1.00 (1.00, 1.01)	0.237	1.00 (1.00, 1.01)	0.475	1.00 (1.00, 1.01)	0.528
**∑Lip-POPs** ⅄	1.00 (1.00, 1.00)	0.974	1.00 (1.00, 1.01)	0.677	1.00 (1.00, 1.01)	0.493	1.00 (1.00, 1.01)	0.542
PFHxS ✦	16.1 (0.19, 1359)	0.220	27.6 (0.23, 3260)	0.173	0.26 (0.00, 29958)	0.263	0.30 (0.00, 131742)	0.304
PFHpS ✦	3.37 (0.00, 184689)	0.827	5.76 (0.00, 320799)	0.754	0.01 (0.00, 4.6E10)	0.755	0.00 (0.00, 6.5E12)	0.725
PFOS ✦	1.03 (0.81, 1.29)	0.830	1.04 (0.82, 1.30)	0.769	0.84 (0.42, 1.68)	0.617	0.66 (0.18, 2.42)	0.525
**∑PFSAs** ✦	1.03 (0.83, 1.28)	0.789	1.04 (0.84, 1.29)	0.729	0.85 (0.44, 1.63)	0.626	0.69 (0.21, 2.25)	0.542
PFOA ✦	0.12 (0.01, 2.16)	0.149	0.06 (0.00, 1.42)	**0.082****	0.02 (0.00, 24.6)	0.255	0.00 (0.00, 6.9E63)	0.580
PFNA ✦	0.96 (0.16, 5.68)	0.964	1.03 (0.19, 5.54)	0.977	0.66 (0.01, 83.1)	0.864	0.42 (0.00, 278.4)	0.794
PFDA ✦	0.95 (0.11, 7.99)	0.965	1.08 (0.13, 9.37)	0.943	1.01 (0.01, 91.2)	0.995	0.57 (0.00, 108.4)	0.834
PFUnA ✦	0.87 (0.43, 1.78)	0.707	0.90 (0.43, 1.89)	0.780	1.03 (0.24, 4.45)	0.964	0.81 (0.18, 3.73)	0.787
**∑PFCAs** ✦	0.87 (0.61, 1.26)	0.475	0.87 (0.61, 1.26)	0.461	0.77 (0.27, 2.18)	0.625	0.73 (0.28, 1.85)	0.501
**∑PFAS'** ✦	0.99 (0.86, 1.14)	0.858	0.99 (0.86, 1.14)	0.889	0.88 (0.58, 1.35)	0.569	0.79 (0.43, 1.44)	0.434
Hg ★	0.99 (0.86, 1.14)	0.892	1.00 (0.88, 1.12)	0.951	0.51 (0.07, 3.80)	0.509	0.33 (0.01, 11.6)	0.539
Pb★	0.94 (0.73, 1.20)	0.611	0.94 (0.72, 1.22)	0.631	1.03 (0.92, 1.16)	0.560	1.06 (0.92, 1.23)	0.413
Cd ★	0.82 (0.14, 4.93)	0.830	1.40 (0.13, 15.7)	0.785	0.29 (0.00, 51.7)	0.639	0.32 (0.00, 709)	0.769
Se ★	1.00 (0.99, 1.01)	0.847	1.00 (0.99, 1.01)	0.901	1.08 (0.99, 1.18)	**0.074****	1.40 (0.00, 2.8E33)	0.993

n: Number of participants in parameter, OR: Odds-ratio, CI: Confidence interval, * Significant (p ≤ 0.050), * * Borderline significant (p ≤ 0.100), Adjustment model 1: Maternal plasma cotinine, maternal educational level, maternal age at delivery, Med = Medium, Cont. = Continuous, n ◊ = n (abnormal), N/A: Test not applicable., ∑LipPOPs = ∑LipophilicPOPs, ⅄ (µg/kg lipid), ✦ (ng/mL), ★ (µg/L)
